# Genomic evidence of environmental and resident *Salmonella* Senftenberg and Montevideo contamination in the pistachio supply-chain

**DOI:** 10.1371/journal.pone.0259471

**Published:** 2021-11-04

**Authors:** Julie Haendiges, Gordon R. Davidson, James B. Pettengill, Elizabeth Reed, Padmini Ramachandran, Tyann Blessington, Jesse D. Miller, Nathan Anderson, Sam Myoda, Eric W. Brown, Jie Zheng, Rohan Tikekar, Maria Hoffmann

**Affiliations:** 1 Center for Food Safety and Applied Nutrition, Food and Drug Administration, College Park, Maryland, United States of America; 2 Department of Nutrition and Food Science, University of Maryland, College Park, Maryland, United States of America; 3 Neogen Corporation, Lansing, Michigan, United States of America; 4 Center for Food Safety and Applied Nutrition, Food and Drug Administration, Bedford Park, Illinois, United States of America; 5 IEH Incorporated, Seattle, Washington, United States of America; Health Directorate, LUXEMBOURG

## Abstract

Pistachios have been implicated in two salmonellosis outbreaks and multiple recalls in the U.S. This study performed an in-depth retrospective data analysis of *Salmonella* associated with pistachios as well as a storage study to evaluate the survivability of *Salmonella* on inoculated inshell pistachios to further understand the genetics and microbiological dynamics of this commodity-pathogen pair. The retrospective data analysis on isolates associated with pistachios was performed utilizing short-read and long-read sequencing technologies. The sequence data were analyzed using two methods: the FDA’s Center for Food Safety and Applied Nutrition Single Nucleotide Polymorphism (SNP) analysis and Whole Genome Multilocus Sequence Typing (wgMLST). The year-long storage study evaluated the survival of five strains of *Salmonella* on pistachios stored at 25 °C at 35% and 54% relative humidity (RH). Our results demonstrate: i) evidence of persistent *Salmonella* Senftenberg and *Salmonella* Montevideo strains in pistachio environments, some of which may be due to clonal resident strains and some of which may be due to preharvest contamination; ii) presence of the Copper Homeostasis and Silver Resistance Island (CHASRI) in *Salmonella* Senftenberg and Montevideo strains in the pistachio supply chain; and iii) the use of metagenomic analysis is a novel tool for determining the composition of serovar survival in a cocktail inoculated storage study.

## Introduction

*Salmonella* has been shown to persist in low-moisture environments and represents a potential hazard for a wide range of low-moisture foods and ready-to-eat products such as nuts, peanut butter, spices, and flour [1]. The ability for *Salmonella* to survive for extended periods of time in these low-moisture products and cause clinical infections is a notable public health concern [2]. Contamination of these products with *Salmonella* can occur pre-harvest, during harvest, during storage, or post-harvest (e.g., after a heat-treatment step) [3].

*Salmonella enterica* subsp. *enterica* serovar Montevideo (*Salmonella* Montevideo) and *Salmonella enterica* subsp. *enterica* serovar Senftenberg (*Salmonella* Senftenberg) have been repeatedly isolated from pistachios dating back to 2008. These isolates were identified during multi-state salmonellosis outbreaks linked to pistachio consumption in 2013 and 2016 [4–8], as well as multiple product recalls across multiple time periods (e.g., 2009, 2010, 2012, 2013, 2016, and 2018) [9, 10]. A previous study noted that as many as six different *Salmonella* serovars (e.g., Agona, Liverpool, Montevideo, Tennessee, Senftenberg, and Worthington) have been isolated from pistachios, and a comparison of the limited number of serovars to the numbers identified in surveys of other tree nuts may suggest a narrow and persistent contamination source [11, 12]. Multiple potential sources of contamination with foodborne pathogens should be considered in regard to the production of produce, including pistachios. Previous land use should be considered in regard to total risk, for example, if the current farmland was known to have previously been used as an animal feeding operation [13]. Adjacent land use may also be a source of potential biological hazards to consider since there is evidence that dust and microorganisms can move a short distance into adjacent almond orchards from an upwind animal operation [14]. Moreover, pathogen-contaminated runoff can occur, and wild animals can leave waste containing pathogens. Although many of these potential sources would concentrate on the orchard floor where there may not be any direct contact with the pistachios due to the use of catch frames during harvest, various farming activities such as cultivation, spraying foliar treatments, and harvest have the potential to aerosolize pathogens and deposit them on pistachios [13].

The United States is the leading producer and exporter of pistachios in the world [15]. Domestically, approximately 99% of the pistachios consumed in the United States are grown in California [16]. In 2018, 264,000 bearing acres of pistachios in California resulted in a total production of 967 million pounds [17]. The harvesting, transportation, wet abrasion (hulling), float tank submersion, drying, and storage of pistachios all include inherent risks for *Salmonella* contamination according to a risk assessment published in 2018 [18]. The researchers were able to model atypical situations and calculate the mean risk of illness from pistachios. In particular, this study showed an increased risk of contamination due to certain pistachio production steps, including use of a float tank and a delay in drying, both of which had the greatest impact on consumer risk [19].

Multistate outbreaks of salmonellosis in 2013 and 2016 linked to contaminated pistachios were investigated by the U.S. Food and Drug Administration (FDA), the Centers for Disease Control and Prevention (CDC), as well as state public health labs [6, 8]. The 2013 outbreak included 8 clinical cases from six states infected with the same strain of *Salmonella* Senftenberg and isolates recovered from pistachio and environmental swabs. The onset of infection was over a 5-month span (January to May 2013), likely owing in part to the long shelf-life of these products. The clinical strains and those recovered from the implicated facility were identified as related by Pulsed Field Electrophoresis (PFGE), producing an identical PFGE pattern. At the time of this outbreak WGS was not routinely performed but in retrospect three clinical isolates, four pistachio isolates, and 17 environmental swabs were sequenced, and the data was uploaded to NCBI. The outbreak in 2016 had a total of eleven documented clinical cases identified by PulseNet (nine cases of *Salmonella* Montevideo and two cases of *Salmonella* Senftenberg), with two hospitalizations in nine states. Onset dates of illnesses from patients covered a 3-month span (January to March 2016). Clinical and food isolates were characterized by PFGE, as well as whole genome sequencing (WGS). FDA reported raw pistachio samples collected at firms supplied by the implicated facility yielded *Salmonella* Montevideo and Senftenberg, while samples of raw pistachios collected during the investigation of the implicated facility yielded *Salmonella* Senftenberg. These *Salmonella* Senftenberg isolates were nearly indistinguishable from the clinical isolates based on WGS results [7].

Next-Generation sequencing (NGS) now regularly allows for further investigation into the genomes of *Salmonella enterica* that are responsible for illnesses, outbreaks, [20–22] and other contamination events, including a determination of whether a resident strain may be causing contamination [23]. Additionally, NGS techniques provide an abundance of data regarding virulence genes, phages, and antimicrobial resistance gene patterns. The ability to capture all this data in one assay that can be used for multiple downstream analyses makes it very appealing. FDA and CDC utilize SNP and cgMLST/wgMLST analysis in the determination of outbreaks of foodborne pathogens [24]. Although these are different methods, the results are very comparable. WGS data are essential to fully characterize a bacterial pathogen of interest and can often assist in finding ways to prevent future contamination [20–22].

In this study, sequence data from *Salmonella* Senftenberg and *Salmonella* Montevideo isolates associated with pistachio outbreaks, recalls, and investigations over a nine-year period (2009–2018) were analyzed to better understand evolutionary relationships among these isolates and, subsequently, to better evaluate their persistence in the pistachio growing and processing environment. In addition to analyzing WGS data, in parallel, we also conducted a storage experiment over a year-long time frame that included the 2016 outbreak strains of *Salmonella* Montevideo and Senftenberg. This latter study focused on the survival rate of these strains on inshell pistachios stored at two humidity levels typical of low moisture foods (a_w_ < 0.7) and further underscored persistence phenotypes among these adapted strains. The novel application of metagenomic sequencing of the cocktail-inoculated pistachios allowed for simultaneous detection of the five different serovars to determine survivability of the strains over the storage period.

## Materials and methods

### Bacterial isolates used for WGS analyses

The isolates used in this study were identified by querying the Pathogen Detection tool (https://www.ncbi.nlm.nih.gov/pathogens/), a publicly available database curated by the National Center of Biotechnology Information (NCBI) [25], that routinely updates genomic sequences from food, environmental sources, and patients. SNP clusters for further analysis were identified based on the following criteria: serovar “Montevideo” or “Senftenberg”, source “pistachio”, and location “United States”. A total of four SNP clusters (two for Senftenberg: PDS000031814, PDS000031739 and two for Montevideo: PDS000027237, PDS000032600) were identified based on our parameters. All isolates found in the SNP cluster associated with pistachios, as well as any other isolates that were closely related within the clade of the NCBI phylogenetic tree, were downloaded for analysis. The metadata (date of collection, source, and location) were also downloaded from NCBI for use in the analysis. The final data set derived from the four SNP clusters consisted of 204 isolates (109 Senftenberg and 95 Montevideo) obtained from clinical, food, and environmental sources; these were sequenced using Illumina technology. The sources of the 109 Senftenberg isolates consisted of almonds (1), chicken (2), clinical (10), environmental swab (43), fertilizer (1), pistachios (51), and tahini (1). These isolates were collected from 1987 to 2018. The sources of the 95 Montevideo isolates used for phylogenetic analysis consisted of almonds (1), raw intact beef (1), cattle (1), chicken (2), clinical (30), environmental swabs (9), blend trail mix (4), missing source (2), mixed nuts (9), pistachios (35), and swine (1). These isolates were collected from 2008 to 2018. Geographically, there were 107 Senftenberg and 89 Montevideo isolates from the United States and 2 Senftenberg and 6 Montevideo from other countries (including Canada, Mexico, and the United Kingdom). The 2013 outbreak investigation isolates (3 clinical, 4 pistachios, and 17 environmental) were found in SNP cluster PDS000031739 (Senftenberg). The 2016 outbreak investigation isolates were contained in SNP clusters PDS000031814 (Senftenberg) and PDS000027237 (Montevideo). There was a total of thirteen pistachio isolates (8 Senftenberg and 5 Montevideo) and 10 clinical isolates (2 Senftenberg and 8 Montevideo) associated with the 2016 outbreak investigation. The complete list of isolates and accompanying metadata can be found in S1 Table. The isolates were sequenced using the Illumina sequencing chemistry by participating GenomeTrakr laboratories [26], CDC PulseNet laboratories, or private labs. Raw reads for all isolates were downloaded from the Sequence Read Archive (SRA) (https://www.ncbi.nlm.nih.gov/sra) using command line tools from the SRA toolkit available on NCBI. *De novo* assemblies were generated with SPAdes v3.13.0 [27] using k-mer lengths of [21, 33, 55, 77, 99, 127], and the options “—careful” and “—only-assembler”.

### *In silico* MLST

Initial sequence analysis of the isolates was performed using an *in silico* MLST approach, based on information available at the *Salmonella enterica* MLST database [28]. Seven loci (*aroC*, *dnaN*, *hemD*, *hisD*, *purE*, *sucA*, and *thrA)* were used for MLST analysis to generate a sequence type (ST).

### Genome closure

Five *Salmonella* Senftenberg genomes and five *Salmonella* Montevideo genomes isolated from pistachios were completely closed using long read sequencing technology for this study (Table 1). These specific isolates were selected as they were sourced from pistachios, readily available in our laboratory, and had different collection dates. Six of the *Salmonella* isolates were sequenced with the PacBio Sequel system (Pacific Biosciences, Menlo Park, CA, USA) and four isolates were sequenced with the Oxford Nanopore GridION (Oxford Nanopore Technologies, Oxford, UK). The ten closed genomes were submitted in DDBJ/EMBL/GenBank and annotated using the NCBI Prokaryotic Genome Annotation Pipeline (PGAP) [29]. Eight of the complete genomes were reported with a detailed method description in two separate genome announcements [30, 31].

**Table 1:** Closed genome statistics from this study.

Sample ID	Serovar	MLST ST	Collection Year	Chromosome/Plasmids Size (bp)	Sequencing Technology	Coverage	GenBank Accessions
CFSAN000258	Montevideo	ST316	2009	4,694,397	PacBio	500×	CP029035
CFSAN045764	Montevideo	ST316	2016	4,694,399	PacBio	375×	CP029039 [30]
CFSAN051296	Montevideo	ST316	2016	4,694,373	PacBio	204×	CP029336 [30]
CFSAN005645	Montevideo	ST138	2009	4,615,193	ONT	103×	CP040380 [25]
CFSAN010209	Montevideo	ST138	2009	4,619,529	ONT	73×	CP040379 [25]
CFSAN000878	Senftenberg	ST14	2009	4,794,849	PacBio	479×	CP029036
pCFSAN000878				319,930			CP029037
CFSAN045763	Senftenberg	ST14	2016	4,766,139	PacBio	275×	CP029038 [30]
CFSAN047866	Senftenberg	ST14	2016	4,807,052	PacBio	477×	CP029040 [30]
FSW0104	Senftenberg	ST185	2013	4,820,913	ONT	77×	CP037894 [25]
CFSAN087304	Senftenberg	ST185	2018	4,819,673	ONT	25×	CP037892 [25]

### Gene-by-gene comparison approach

To provide gene-by-gene comparison, we aligned all of our closed genomes based on serovar using MAUVE aligner version 20150226 using the progressive algorithm with default settings, and phages were identified using PHASTER [32]. Any unique genomic regions, phages, and plasmids identified were imported into Ridom SeqSphere^+^ v. 6.0.2 (Ridom GmbH, Munster, Germany) [33] and used as a reference to interrogate the 201 short read isolate assemblies used in this study for presence or absence.

### Phylogenetic analyses (wgMLST and SNP)

Targeted core genome MLST (cgMLST) and accessory genome analysis of the isolates was performed using Ridom SeqSphere^+^ software v 6.0.2 [33] by creating *ad hoc* wgMLST databases derived from the closed genomes previously described. Using the cgMLST target definer tool and accessing our genomes from NCBI, an *ad hoc* wgMLST scheme for *Salmonella* Senftenberg was developed. The scheme was based on the closed reference genome CFSAN047866 (CP029040), comprised of 4,768 genes, and query genomes (CP029036, CP029038, CP037892, and CP037894) found in Table 1 using the thresholds previously published [34]. The resulting scheme consists of 4,548 loci (3,908 core targets and 640 accessory targets). The 109 *Salmonella* Senftenberg assemblies (54 nuts/seeds, 44 environmental, 10 clinical and 1 poultry) were typed using this scheme.

The same process was repeated to create an *ad hoc* wgMLST scheme for *Salmonella* Montevideo using the closed genome CFSAN051296 (CP029336), comprised of 4,664 genes, as the reference and the four other closed genomes from this study (CP029035, CP029039, CP040379, and CP040380) as query genomes using the same settings to define the core genome loci. The resulting scheme consists of 4,447 loci (3,876 core targets and 571 accessory targets). The 95 *Salmonella* Montevideo assemblies (49 nuts/seeds, 30 clinical, 9 environmental, 2 poultry, 2 missing source, 1 beef, 1 swine, and 1 cattle) were typed using this scheme.

For each serovar, the alleles identified were used to establish a matrix disregarding missing allele values. A minimum spanning tree of all common alleles was then constructed to visualize the results using the Ridom SeqSphere^+^ software. Clusters were automatically assigned using the default distant threshold for the software of ≤ 10 alleles.

Due to the large allele differences identified in the wgMLST analysis, phylogenetic SNP analysis was performed separately on the four different sequence types of *Salmonella* Senftenberg and Montevideo using the CFSAN SNP Pipeline [35]. In short, WGS reads were mapped to the reference genome using Bowtie2 and the resulting files were processed using SAMtools. The variant sites were identified using VarScan, and a SNP matrix was produced using custom scripts. Four SNP matrices were generated using the closed genomes CFSAN000878 (ST14), FSW0104 (ST185), CFSAN000258 (ST316), and CFSAN005645 (ST138) as references. The SNP matrices can be found in S2–S5 Tables. Phylogenetic trees were constructed using the RAxML (Randomized Accelerated Maximum Likelihood) program with 500 bootstrap replicates [36]. All RAxML analyses were performed with the default parameter settings and the GTRCAT nucleotide substitution model. The trees were visualized using Figtree software (version 1.4.4).

### Pistachio storage study

A storage study was conducted to evaluate the ability for *Salmonella* (including *Salmonella* Senftenberg and Montevideo isolates associated with the 2016 outbreak) to persist on raw, inshell pistachios in conditions simulating a range of typical storage conditions. A cocktail containing four different *Salmonella enterica* serovars (Anatum, Montevideo, Oranienberg, and Senftenberg) associated with low moisture food commodities and one strain of *Salmonella* Newport isolated from tomatoes was used in this experiment (Table 2). The genomes of all five isolates were completely closed using Pacbio long read technology [30, 37]. For this study, organic, raw inshell pistachios were purchased and tested for background microbial populations using the Biomerieux Tempo AC kit (Biomerieux, Durham, NC, USA), and then inoculated with the 5-strain cocktail. Inoculum was prepared from stock culture as described in Keller et al. [38] with slight modifications. Briefly, a single colony from each working stock was transferred to Trypticase Soy Broth (TSB, BD, Franklin Lakes, NJ, USA) and incubated overnight at 37°C. After incubation, 100 μl of inoculum was spread plated on Trypticase Soy Agar (TSA, BD) plates. The plates were incubated overnight at 37°C and then bacteria were harvested by adding 1.0 ml of Buffered Peptone Water (BPW, BD) to the surface of the plate and gently scraped using an L-shaped plate spreader. Each plate yielded ~0.5 ml of harvested culture at approximately 10 log CFU/ ml. The stock cocktail was formed by combining equal volumes of all five harvested cultures and then a high-population cell suspension was prepared by combining 18 ml of the five-serotype *Salmonella* stock cocktail with 1,800 ml of sterile deionized water. A total of 900 g of pistachios were soaked in the high-population cell suspension bath for 1 minute, and then placed on trays lined with absorbent towels in a biosafety cabinet to airdry overnight at room temperature. After 24 h, pistachios were stored in desiccator cabinets equilibrated to either 35% relative humidity (RH) or 54% RH at 25°C, which were selected to represent a low and high water activity (a_w_) within the range of water activity levels typical of low moisture foods [39]. Uninoculated control pistachios were held under the same conditions. To determine the surviving populations, triplicate (10g) samples were removed for analysis at 0, 1, 2, 4, 6, 13, 27, 55, 83, 180, 270, and 365 days. The water activity was recorded for the inoculated and control pistachios from the different relative humidity conditions using an Aqualab model 4TE water meter (Decagon Devices, Pullman, WA). Two random 10 g samples from the uninoculated control group were tested using the Biomerieux Tempo AC kit to determine background microbe counts. The triplicate samples of inoculated pistachios were diluted 1:9 in BPW and appropriate dilutions were plated in duplicate on the differential media m-TSAYE [Trypticase Soy Agar with Yeast Extract supplemented with 0.05% (wt/vol) ammonium iron citrate and 0.03% sodium thiosulfate]. After overnight incubation at 37°C, colonies were counted, and log CFU/g was calculated. A repeated measure analysis of variance (ANOVA) was performed in R v3.6.1 to determine the differences in log CFU/g over time between the high and low humidity conditions. The differences in the slopes (or rate of reduction in log CFU/g) between the two humidity conditions were also investigated using R.

**Table 2:** Strains used in storage study to assess persistence in pistachios.

Strain	Serovar	Source	MLST (ST)
CFSAN076215	Anatum	Peanuts	64
CFSAN051296	Montevideo	Pistachio	316
CFSAN000836	Newport	Tomato	118
CFSAN076211	Oranienburg	Pecans	3613
CFSAN045763	Senftenberg	Pistachio	14

Two methods were used to determine composition of surviving serovars at different time points, the Luminex xMAP^®^ *Salmonella* Serotyping Assay (Luminex, Madison, WI, USA) and metagenomic analysis of shotgun sequencing using the Illumina MiSeq benchtop sequencer (Illumina, San Diego, CA, USA). For the Luminex assay, 12–16 individual colonies from each condition were selected from the m-TSAYE plates and DNA was extracted using Bio-Rad InstaGene^™^ matrix (Bio-Rad, Hercules, CA) following the manufacturer’s protocol for DNA extraction from bacterial cells. DNA was analyzed for molecular determination of serotype in *Salmonella* following the protocol published by the CDC [40, 41]. For the sequencing assay, the initial 1:9 dilution of pistachio:BPW was incubated at 37°C overnight. A 2 ml aliquot of the overnight enrichment was centrifuged at 6000 RPM for 10 minutes and then DNA was extracted using the Maxwell RSC Cultured Cells DNA Kit (Promega, Madison, WI, USA) according to manufacturer’s recommendations with the addition of RNase A after lysis. Sequencing libraries were prepared using Nextera DNA Prep Library kit (Illumina, San Diego, CA) following the manufacturer’s protocols. The finished libraries were sequenced on the Illumina MiSeq using v3 600-cycle sequencing chemistry with 2 × 250 paired end reads. Determination of serovar composition from shotgun sequencing raw reads was conducted using custom C++ programs developed to compile a k-mer signature database containing multiple unique 30 bp sequences per species and then identification of each read in the input file using the 30 bp probes [42]. For each bacterial species or subspecies, each non-duplicated 30-mer from a reference whole genome sequence was placed into a database. We removed any k-mers not found in at least 2/3 of a set of additional genome sequences of the same species and we removed any k-mer found in genomes of other species. The resulting k-mer database used in this work contains 5,900 target entries, each consisting of approximately 40,000 (range 44 to 80,000) unique k-mers. The database includes 1,100 different bacterial genera, and 3,500 species. Normalization is performed to correct for bias due to differing number of k-mers used per database entry and the results are tabulated as percent of identified reads (contribution to the microbial population of identified species) for each database entry.

## Results and discussion

### *In silico* MLST

*In silico* seven locus MLST was performed on the 109 *Salmonella* Senftenberg and 95 Montevideo isolates identified from the Pathogen Detection browser. MLST results for the *Salmonella* Senftenberg isolates identified two different STs. The 54 isolates from NCBI SNP cluster PDS000031814 belong to ST14, while the 55 isolates from SNP cluster PDS000031739 belong to ST185. The ST14 group includes seven of the clinical isolates and 39 isolates from pistachios with a date of collection ranging from 2009 to 2016. The ST185 group contains three clinical isolates and 12 isolates from pistachios collected between 2013 and 2018.

The MLST results for *Salmonella* Montevideo isolates also detected two STs. SNP cluster PDS000027237 is a member of ST316, while SNP cluster PDS000032600 belongs to ST138. The ST316 group contains a total of 82 isolates, of which 24 were clinical isolates and 33 were isolates from pistachio sources ranging in collection date from 2009 to 2018. ST316 has previously been described as the “outbreak” clade due to its linkage with numerous outbreak-associated plant products including tahini, sprouts, spices, as well as pistachios [43]. The ST138 group of 13 isolates contains 6 clinical isolates and 3 isolates from pistachios. The ST results can be found in S1 Table and clearly indicate that pistachios have been contaminated by multiple strains of *Salmonella* Senftenberg and Montevideo.

### Comparative analysis of closed genomes

Long Read Sequencing technology was employed to generate 10 closed genomes for 2–3 representatives of each ST to use as a reference for SNP analysis, as well as to identify the positions of mobile elements and assess genetic differences between STs. The *Salmonella* Senftenberg isolates ranged from 4.7–4.8 mb in size while the *Salmonella* Montevideo isolates were ~ 4.6 mb. One plasmid, over 300 kb in size, was found associated with *Salmonella* Senftenberg CFSAN000878. The genome statistics for these ten isolates can be found in Table 1.

**Fig 1:**
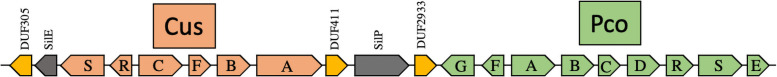
Copper and Silver Resistance Island (CHASRI) diagram. Structure of the CHASRI genes in our closed genomes. Protein accessions for CP029039: *pcoE* (AWE27745.1), *pcoS* (AWE27744.1), *pcoR* (AWE27743.1), *pcoD* (AWE27742.1), *pcoC* (AWE27741.1), *pcoB* (AWE27740.1), *pcoA* (AWE27739.1), *pcoF* (AWE27738.1), *pcoG* (AWE27737.1), DUF2933 (AWE27736.1), *silP* (AWE27735.1), DUF411 (AWE27734.1), *cusA* (AWE27733.1), *cusB* (AWE27732.1), *cusF* (AWE27731.1), *cusC* (AWE27730.1), *cusR* (AWE27729.1), *cusS* (AWE27728.1), *silE* (AWE27727.1), DUF305 (AWE27726.1).

The content of the ten closed genomes were compared to each other to find and identify genomic elements that a clonal strain may acquire or dispose of over time. The closed genomes were evaluated for conserved genes shared within the same sequence type, as well as between both serovars (Senftenberg and Montevideo). As expected, the isolates within the same sequence type showed higher similarity. Interestingly, one unique region was identified to be shared between the ST14 and ST316 closed genomes but missing in the ST185 and ST138 genomes. This region was determined to be the Copper Homeostasis and Silver Resistance Island (CHASRI) (Fig 1) and is ~ 21 kb in size [44]. Based on analysis using Ridom SeqSphere^+^, this island was confirmed in all of the short-read sequence data of isolates associated with ST14 and ST316 and was not present in the ST185 or ST138 isolates. The island is located on the chromosome in both serovars and contains the genes *pco* operon, *cus* operon, *silE*, and *silP*. The sequence of the CHASRI had a 100% identity intra-serovar and a 99.95% identity inter-serovar when the region was analyzed with BLAST [45]. The CHASRI has been identified previously in *Escherichia coli*, *Enterobacter cloacae*, *Klebsiella pneumoniae*, and *Salmonella enterica* [44]. This island has previously been shown to increase the ability of a bacterium to survive in an environment with higher levels of copper and silver, as well as the ability to provide tolerance during the transition between aerobic and anaerobic environments, affording a fitness advantage for facultative anaerobes [44, 46]. The use of copper as a growth promoter in swine and poultry has led to the selection of this island in *Salmonella* associated with these sources in other countries [47, 48]. The use of copper as a fungicide and bactericide in agriculture has been reported for many years, and it is generally considered safe for both conventional and organic produce use [49, 50]. Copper deficiency is reportedly common in pistachios and the use of copper in foliar and soil treatments has been recommended to the industry [51]. Copper-containing foliar sprays may be applied at various times while pistachios are developing in the trees [52]. The presence of the CHASRI in the pistachio-associated isolates may be an acquired adaptation by these specific salmonellae in response to copper stress in the growing environment.

The IncH12/IncH12A plasmid (CP029037) identified in *Salmonella* Senftenberg CFSAN000878, with a length of 319,930 bp, contains multiple heavy metal resistance genes which confer arsenic resistance (*arsB*, *arsC*, *arsH)*, mercury resistance (*merA*, *merC*, *merD*, *merP*, *merR*, *merT)*, and tellurium resistance (*terABCDEF*), as well as a copy of *silE*, *cusA*, and *cusS*. The plasmid also contains one antimicrobial resistance gene, MCR-9.1, which confers Colistin resistance and is of significant public health importance [53, 54]. The plasmid sequences for the duplicated genes, *silE*, *cusA/czcA*, *and cusS*, differ from the same genes found in the chromosome with 91.2%, 93.7%, and 95.1% identity, respectively. This plasmid was only identified in one other isolate (CFSAN016599) from pistachio, also collected in 2009. Since the plasmid has not been identified in any isolates after 2009, it is plausible that it was lost over time due to its large size and the genes on this plasmid not being necessary but rather redundant for increased fitness due to the incorporated CHASRI already in the chromosome.

Phages can encode factors that increase the virulence of *Salmonella* by enhancing adhesion, intracellular survival, and host entry [55]. Phages were identified using PHASTER, and those sequences were then used as references in Ridom SeqSphere^+^ to interrogate the short-read assemblies. PHASTER identified a unique intact phage, Salmon_SPN3UB_NC019545, which was found in one of the closed genomes from ST14. Specifically, this phage was found in the closed genome of CFSAN047866 (CP029040) as well as in the short-read data from 7 of 54 isolates by analysis with Ridom SeqSphere^+^. Interestingly, these isolates were not associated with a single group on the SNP tree. Phages identified in the other sequence types were found in 98–100% of all isolates, and the phages identified can be found in S6 Table.

### Phylogenetic analyses using wgMLST and SNPs

Whole genome multi-locus sequence typing (wgMLST)-based methods have been previously employed for analysis of *Salmonella enterica* outbreaks and clusters [34, 56] and are less computationally expensive in comparison with SNP analysis. An advantage of using an *ad hoc* wgMLST approach is the ability to analyze isolates without the need for a reference sequence. In this study, two *ad hoc* wgMLST schemes were created for analysis of the *Salmonella* Senftenberg and *Salmonella* Montevideo isolates. In addition to the wgMLST analysis, the CFSAN SNP pipeline [35] was utilized to perform a reference-based variant detection analysis which is compatible for isolates that are closely related and allows for higher resolution of these isolates. This application should only be used on isolates that belong to the same NCBI SNP cluster [57], therefore the pipeline was utilized here to produce four individual SNP matrices for the different STs.

#### *Salmonella* Senftenberg

**Fig 2:**
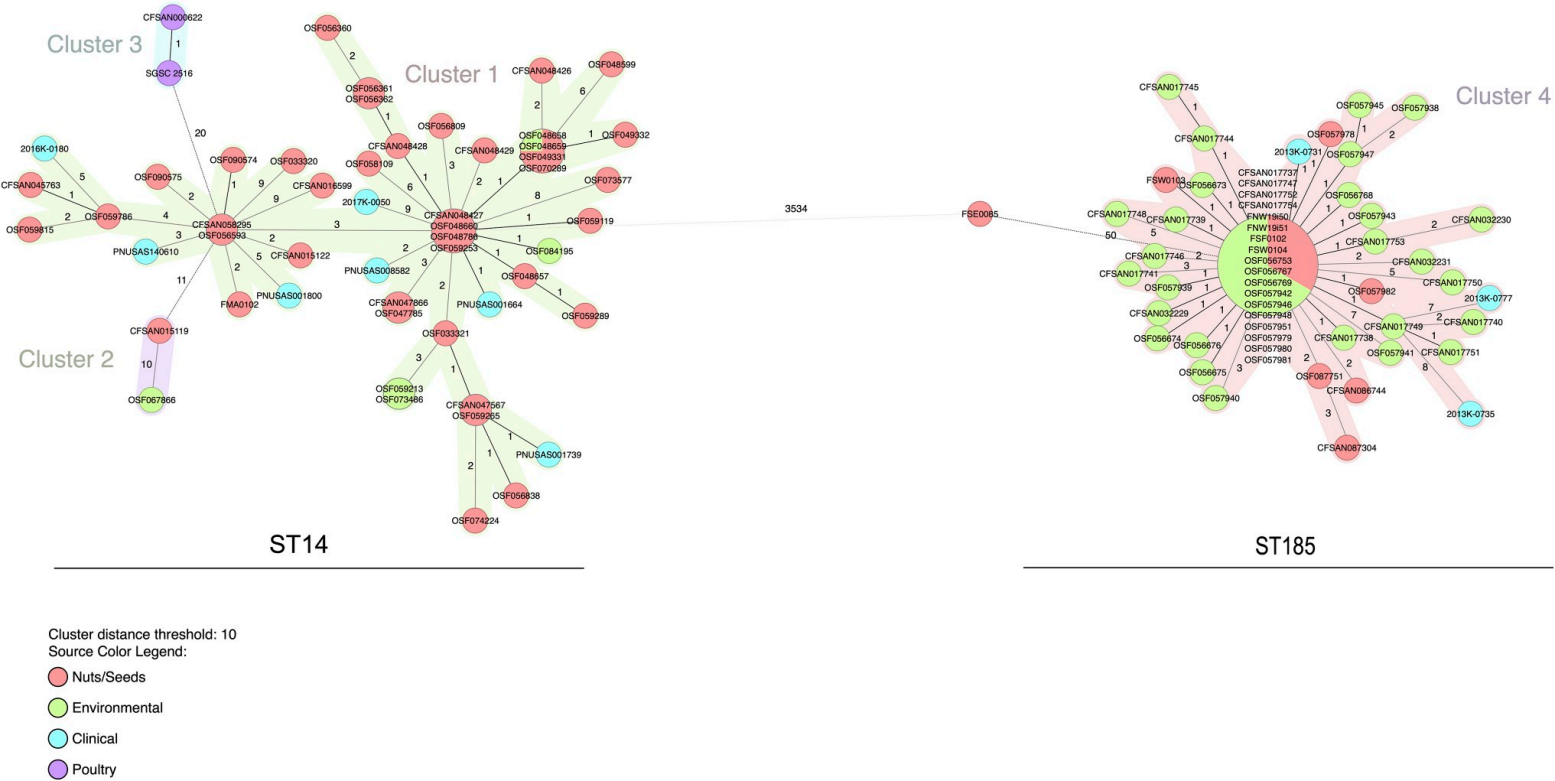
Minimum spanning tree of the wgMLST allelic profiles for *Salmonella* Senftenberg isolates (n = 106) using Ridom SeqSphere^+^. Distance based on wgMLST typing of 3,891 common loci (3,591 core genes and 300 accessory genes) using the parameters “pairwise ignoring missing values” during calculation. Isolates with 0 allele difference are found in the same node. Allele distances between samples are indicated, clusters are defined with maximum 10 alleles distance. Samples are color coded by their isolate source as in the legend.

A minimum spanning tree produced by analysis of all common allele calls from both the core genome and accessory genome shows that *Salmonella* Senftenberg isolates in ST14 are distinct from isolates in ST185 by a difference of 3,534 alleles (Fig 2). This allele difference between ST14 and ST185 is meaningful and illustrates that the primary contamination sources for these isolates are not the same. The analysis separated the isolates belonging to ST14 into three clusters (Clusters 1, 2, and 3) and the ST185 isolates into one cluster (Cluster 4). Cluster 1 contains the majority of the ST14 isolates, including those isolates associated with the 2016 outbreak. The pistachio isolates from ST14 have more branching and allele difference (range of 0–11) over an eight-year period (2009–2017). All the ST185 isolates associated with pistachios including the 2013 outbreak isolates are located in Cluster 4 and have low allele differences (0–7) over a five-year period (2013–2018), illustrating genome conservation and clonality.

**Fig 3:**
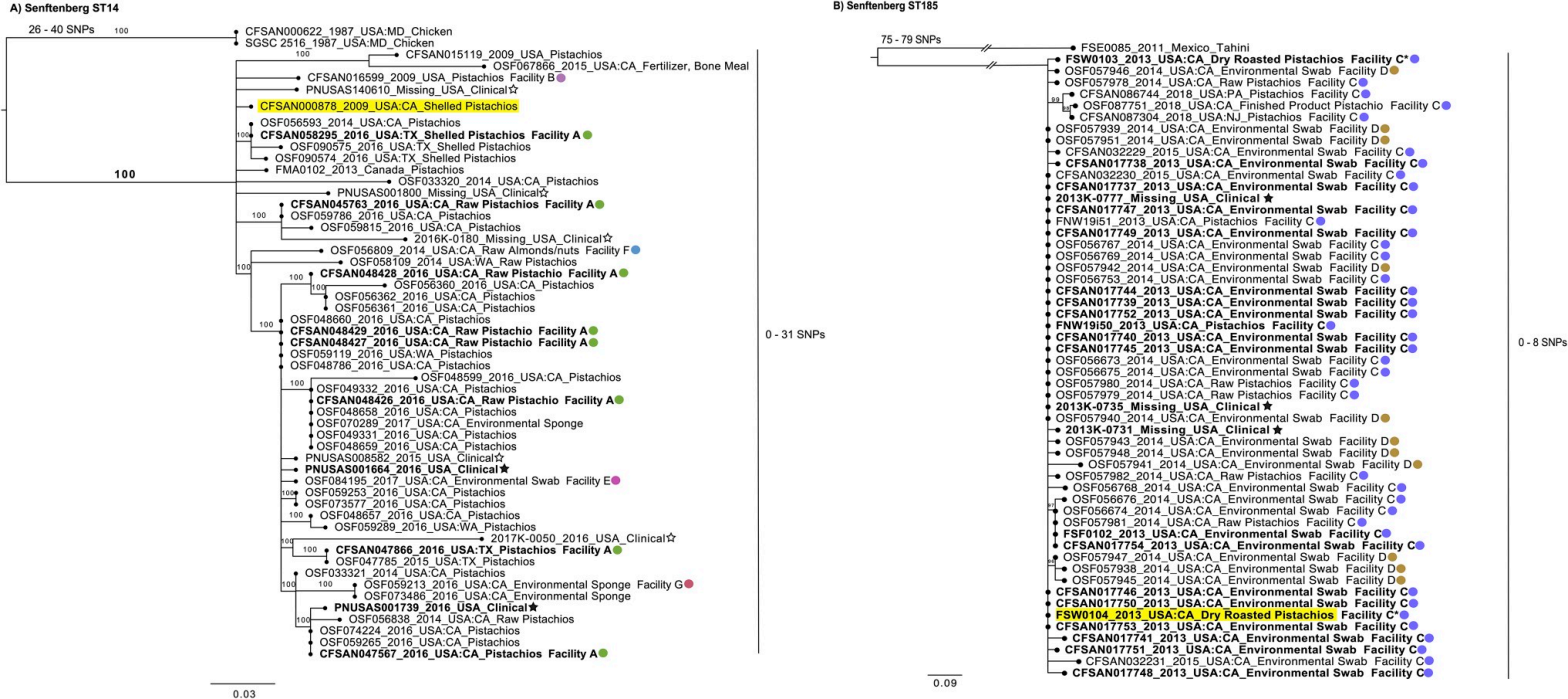
Phylogenetic analysis based on SNPs found in the *Salmonella* Senftenberg strains of this study. A SNP matrix was generated for both sets of isolates based on ST with the CFSAN SNP Pipeline [35]. The SNP matrix was analyzed using RAxML using the GTRCAT substitution model and 500 bootstrap replicates. Reference strains are highlighted in yellow. The outbreak associated isolates are in bold. Clinical isolates have a star symbol; black star = outbreak associated. Facility identifiers are also highlighted on the tree with different color circles. A) Maximum likelihood tree based on SNPs found in the 54 isolates from the ST14 group. B) Maximum likelihood tree based on SNPs found in the 55 isolates from the ST185 group.

The reference-based SNP analysis of the 55 *Salmonella* Senftenberg isolates that are typed as ST14, using CFSAN000878 as the reference, identified 146 variant positions and a maximum likelihood tree was constructed using RAxML (Fig 3A). Anonymized facility identifiers were used for those isolates where the source facility was known. The pistachio isolates from 2009–2017 share a common ancestor, and do not form distinct lineages based on the year of isolation. The isolates, both clinical and those associated with pistachios from the 2016 outbreak investigation, have a median of 4 SNPs and a range of 0–9 SNPs. The outbreak investigation associated isolates appear throughout the tree, suggesting that multiple subpopulations of *Salmonella* strains are potentially contaminating pistachios. Akin to this observation, five other clinical isolates, that are not associated with the 2016 outbreak, have relatively close relationships to pistachio isolates. For instance, PNUSA008582 is 1 SNP away from both CFSAN048429 and CFSAN048427, while the other four clinicals have a minimum SNP distance of 5–13 SNPs from the nearest 2016 pistachio outbreak strain. The main clade has a total diversity range of 0–31 SNPs with a median of 7 SNPs. Previous research has shown SNP differences in *Salmonella* outbreaks to be <21 SNPs [21, 26, 58–60] with an outlier in serovar Agona with 30 SNP differences [61]. From our study, a 2015 fertilizer (bone meal) isolate, OSF067866, was noted to be 9 SNPs distant from a 2009 pistachio isolate, CFSAN015119. While a definitive linkage of these two isolates is unattainable, it is notable that the use of pistachio and almond hulls as a soil amendment has been explored [62–66]. Additional examination of soil amendments and fertilizer blends containing tree nut hulls may shed additional light on potential reservoirs and persistence of *Salmonella* in orchard soil. The results from both wgMLST and SNP analysis were comparable and are indicative of *Salmonella* residence in the pistachio primary production environment (i.e., orchard) for isolates belonging to ST14. Further evidence supporting this hypothesis of *Salmonella* residence in the orchard environment is that there are isolates collected from five additional facilities (including pistachio and environmental sources), located in California, and not linked to the 2016 outbreak, over multiple years are found throughout the SNP tree. Finally, the finding that all the ST14 isolates contain the CHASRI island–coinciding with the known use of copper in pistachio orchards which may be providing selective pressure for this adaptation, as well as previous studies showing poultry isolates containing this island–adds support that these strains have established residence in or around the production environment. Although, the initial source of the environmental contamination (e.g., adjacent animal operations, use of biological soil amendments of animal origin (BSAAO), water, or wildlife) of this strain cannot be determined, our analysis shows that it was able to establish residence.

For the second Senftenberg SNP tree, closed genome FSW0104 was used as the reference genome to analyze the 55 isolates identified by MLST to belong to ST185 (Fig 3B). The SNP analysis identified 109 variant positions within these isolates. The isolate FSE0085, tahini from Mexico, was the closest related isolate (75–79 SNPs) to the rest of the isolates (n = 54) which grouped together to form a single monophyletic clade. The 51 pistachio and environmental swab isolates in this clade were collected from two different facilities, Facility C and Facility D, both located in California. The 2013 outbreak isolates (clinical, pistachio, and environmental swabs) have a median 1 SNP distance and a range of 0–3 SNPs from each other. This outbreak investigation also includes two isolates of roasted pistachios (FSW0103 and FSW0104) from Facility C that were shipped to another facility (Facility C*). This outbreak investigation resulted in identification of deficiencies in Good Manufacturing Practices (GMPs) at Facility C, which may have contributed to the outbreak [8]. This strain has not only been associated with the 2013 outbreak but repeated isolation from pistachios has resulted in additional product recalls. For example, the pistachio isolates collected in the 2018 recall form a group that is a median of 4 SNPs distant (range 3–8 SNPs) from those collected in 2013, 2014, and 2015. Both phylogenetic analyses indicate genome conservation and clonality with very little variability between the isolates from these two facilities (C and D). The SNP analysis yields a median of 1 SNPs distance and a range of 0–8 SNPs over a five-year period and the wgMLST results illustrates a distinct central node within the ST185 isolates with low allele differences (0–7). The minimal genetic differences of these isolates is indicative of a persistent strain that is not under stress and experiencing slower than normal evolutionary events [67]. The 2013 outbreak investigation illustrated how the same strain can move between facilities (C and C*). Therefore, some theoretical explanations for the presence of *Salmonella* isolates from Facility C and D with 0 SNP and allele distances between them include: a) *Salmonella* establishing residence in the facilities after both receiving pistachios contaminated by a possible shared grower, harvester, or transporter in or before 2013 or b) a business relationship that allows for pistachios or equipment to be shared or exchanged between the two facilities. It seems unlikely that two strains with no SNP/allele differences would occur in disparate facilities without some type of indirect or direct relationship between them. Rather, the small number of genetic differences over an extended period would suggest the transmission of the same strain between the two facilities and therefore a clonal strain taking harborage [23, 68]. Furthermore, the lack of any measurable SNP distance between the *Salmonella* isolates from these two facilities suggests that such residence would be in a setting with relatively low environmental stress, such as an environment devoid of implementation of more robust sanitation controls.

#### *Salmonella* Montevideo

**Fig 4:**
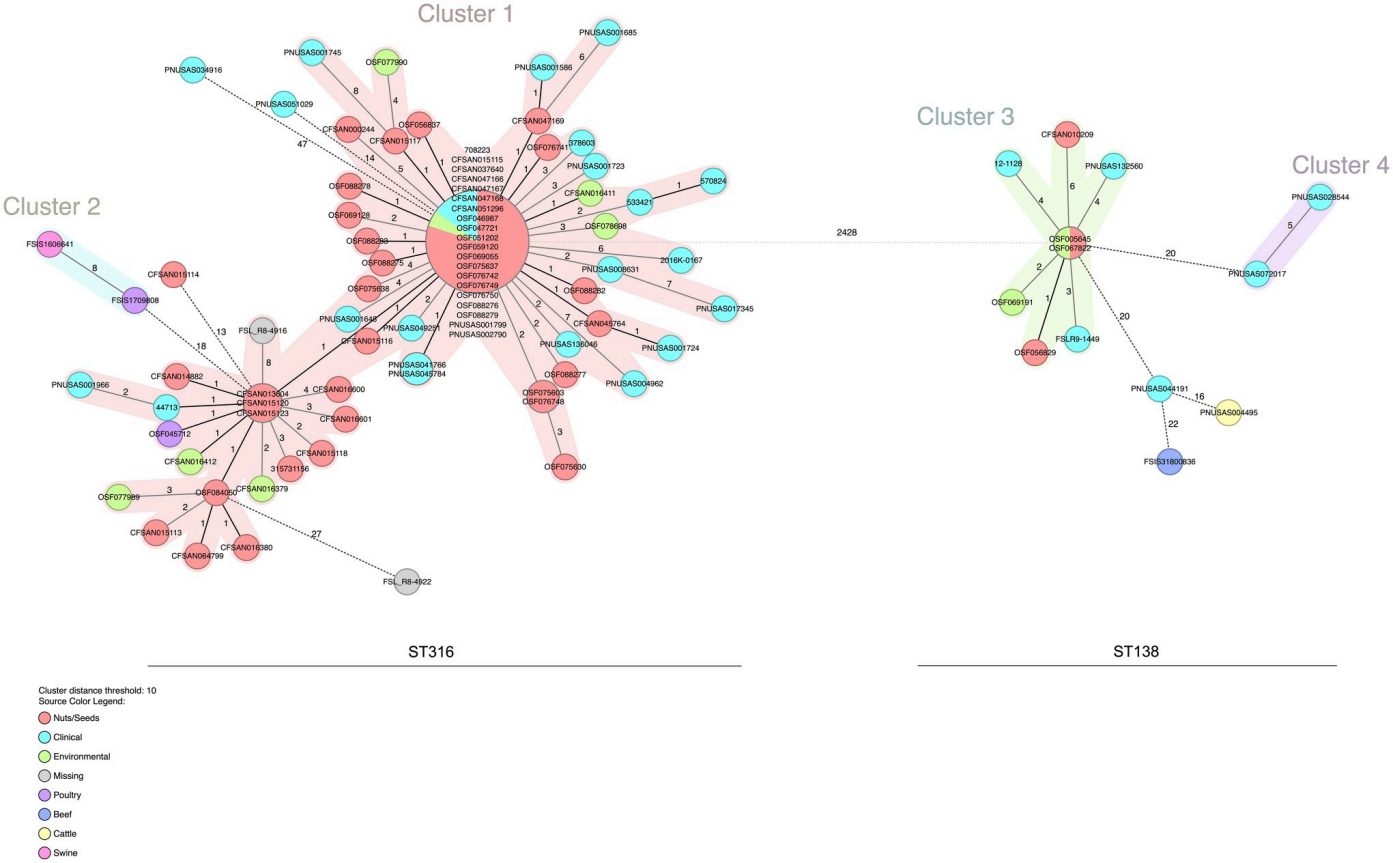
Minimum spanning tree of the wgMLST allelic profiles for *Salmonella* Montevideo isolates (n = 95) using Ridom SeqSphere^+^. Distance based on wgMLST typing of 3,876 common loci (3,634 core genes and 242 accessory genes) using the parameters “pairwise ignoring missing values” during calculation. Isolates with 0 allele difference are found in the same node. Allele distances between samples are indicated, clusters are defined with maximum 10 alleles distance. Samples are color coded by their isolate source as in the legend.

A minimum spanning tree produced by distance calculation for all common allele calls from both the core genome and accessory genome shows a distinct difference of 2,428 alleles between the *Salmonella* Montevideo isolates from ST316 and ST138 (Fig 4). Similar to Senftenberg isolates, these isolates also possess a sizeable allele disparity between the sequence types, which would be indicative of different sources of the contamination. Four well-defined clusters were identified, two for each ST (ST316 –Cluster 1 and 2, ST138 –Cluster 3 and 4). A majority of the isolates from pistachios are in a single cluster for each ST (Cluster 1 and Cluster 3), whereas those isolates associated with the 2016 outbreak are in Cluster 1. A single isolate from trail mix (CFSAN015114–19 alleles) collected in 2008, as well as two clinical isolates (PNUSAS034916–47 alleles and PNUSAS051029–14 alleles) were found to fall outside of Cluster 1. Interestingly, five clinical isolates from the United Kingdom were found in proximity to the outbreak Cluster 1 of pistachio isolates (0–4 alleles) (Fig 3). One of these isolates was collected prior to the outbreak in 2014, and four were collected after the outbreak (2017–2019). There are two clinicals, PNUSAS041766 and PNUSAS045784, that are a match to each other and are one allele from the large node of outbreak-associated isolates. The ST138 pistachio isolates are found in Cluster 3. Three clinical isolates with 3–4 allele differences from an ST138 pistachio isolate are in Cluster 3 with one of these isolates, 12–1128, located in Canada.

**Fig 5:**
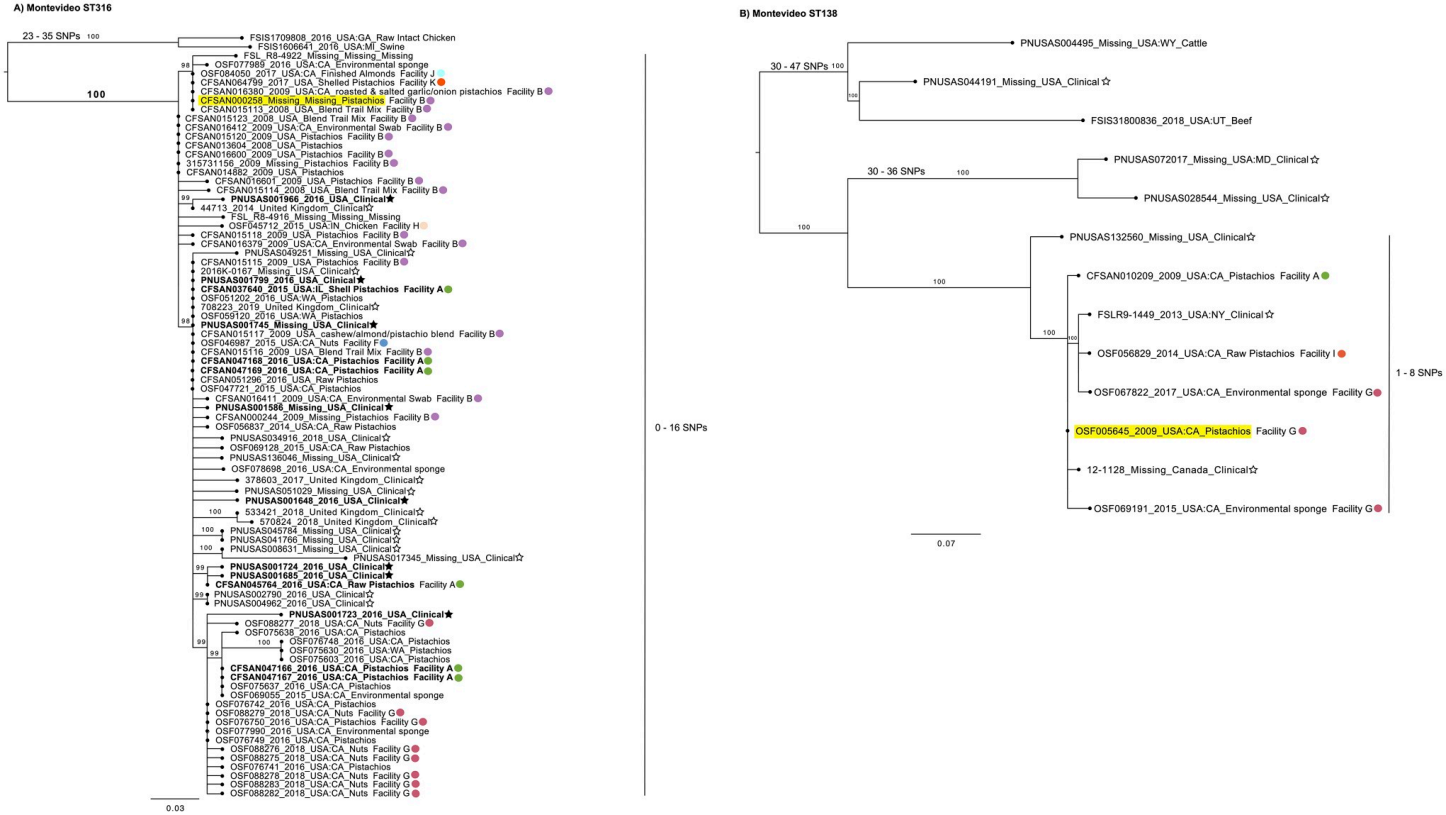
Phylogenetic analysis based on SNPs found in the *Salmonella* Montevideo strains of this study. A SNP matrix was generated for both sets of isolates based on ST with the CFSAN SNP Pipeline [35]. The SNP matrix was analyzed using RAxML using the GTRCAT substitution model and 500 bootstrap replicates. Reference strains are highlighted in yellow. Isolates associated with the 2016 outbreak are in bold font. Clinical isolates have a star symbol; black star = outbreak associated. Facility identifiers are also highlighted on the tree with different color circles A) Maximum likelihood tree based on SNPs found in the 82 isolates from the ST316 group. B) Maximum likelihood tree based on SNPs found in the 13 isolates from the ST138 group.

Reference-based SNP analysis identified a total of 107 distinct SNP variants in the isolates of ST316 using CFSAN000258 as the reference genome. A resultant phylogenetic tree, capturing the relationship among the 82 isolates belonging to ST316, yielded several important findings (Fig 5A). Isolates related to the 2016 outbreak are not associated with one specific lineage on the tree. Isolates from seven different facilities can be found throughout the tree. The paraphyletic grouping of the clade breaks down into smaller subclades, with a total SNP distance ranging from 0–16 SNPs with a median of 3 SNPs. The 2016 outbreak strains have a median distance of 2 SNPs (range 0–10 SNPs). As observed in the wgMLST analysis, other non-epidemiologically linked clinical isolates from the United States and the United Kingdom were found to be closely related to pistachio isolates, with a minimum SNP distance range of 0–11 SNPs and 0–4 SNPs, respectively. With the continued rise in imported/exported goods, the observation of the large number of clinical isolates that are clustered near pistachio isolates from both the United States and the United Kingdom further highlights the benefit of global partnerships in the area of food safety and public health and the need for increased metadata inclusion and transparency [69].

The presence of a chicken isolate (OSF045712) from Facility H located within the California nut clade can possibly be explained by the use of a tree nut supplemented animal feed [70–72]. This scenario is supported by domestic pistachio firms advertising the distribution of their hulls for animal feed to promote sustainability [73, 74]. The chicken isolate is 2–5 SNPs away from other tree nut isolates in the clade. This potential connection between a contaminated component of animal feed and livestock that eventually enters the food supply for humans illustrates the interconnectedness of many food production systems and the importance of a “One Health Approach” [75] for food safety. Akin to the Senftenberg ST14 group, the CHASRI is identified in each of these isolates. The combined findings of CHASRI presence, the increased changes occurring over eight years, and that the isolates are derived from seven different facilities are consistent with the hypothesis of a persistent strain in the pistachio production environment that is most likely co-residing with *Salmonella* Senftenberg.

A phylogenetic tree was constructed for the 13 Montevideo isolates from ST138 (Fig 5B) based on a total of 48 distinct SNP variants identified by reference-based SNP analysis using CFSAN010508 as the reference genome. The three clinical isolates are within 1–8 SNPs (median 3 SNPs) from the pistachio isolates from three different facilities (Facility A, Facility G, and Facility I), however, without epidemiological data we are unable to positively link these illnesses with contaminated pistachio exposure. Three isolates from Facility G, two environmental swabs and one pistachio, also belong to this clade. The SNP distance between the strains from Facility G (isolated in 2009 (OSF005645), 2015 (OSF069191), and 2017 (OSF067822)) is only 2 SNPs. The wgMLST shows this same relationship with 0 allele differences between OSF005645 and OSF067822 and 1 allele difference to OSF056829. This observation points to substantial conservation within the genome over a 5-year period. The pistachio and swab isolates from three unrelated facilities (Facility A, Facility G and Facility I), that were collected over a wide timespan (8 years) with low diversity, suggests a single contamination event with a persistent *Salmonella* strain. These strains are likely to reside in a lower-stress environment than in the orchard environment, due to the low SNP/allele distances suggest a strain that is not undergoing selective pressure and may also have a slower generation time similar to a resident pathogen [23]. Possible low-stress environments could include shared farm equipment, transport vehicles, or an intermediate space such as silos.

### Storage study to assess persistence in pistachios

To further explore the nature and likelihood of long-term environmental persistence of these *Salmonella*, we tested the survival of *Salmonella* strains in a cocktail after inoculation on raw inshell pistachios. The strains used in this study were associated with low-moisture food products (peanuts (Anatum), pecans (Oranienburg), pistachios (Montevideo and Senftenberg)) and a strain isolated from tomato (Newport) (Table 2). The Senftenberg and Montevideo strains used contain the CHASRI and are from ST14 and ST316, respectively (Table 2). The basis behind using the Newport strain was to determine if there were differences in survivability on a low-moisture food based on the initial source of the isolate (i.e., a high-moisture food). Pistachios were inoculated with a five-serovar cocktail of *Salmonella enterica* and stored at one of two different relative humidity levels (35% RH and 54% RH) at 25°C.

**Fig 6:**
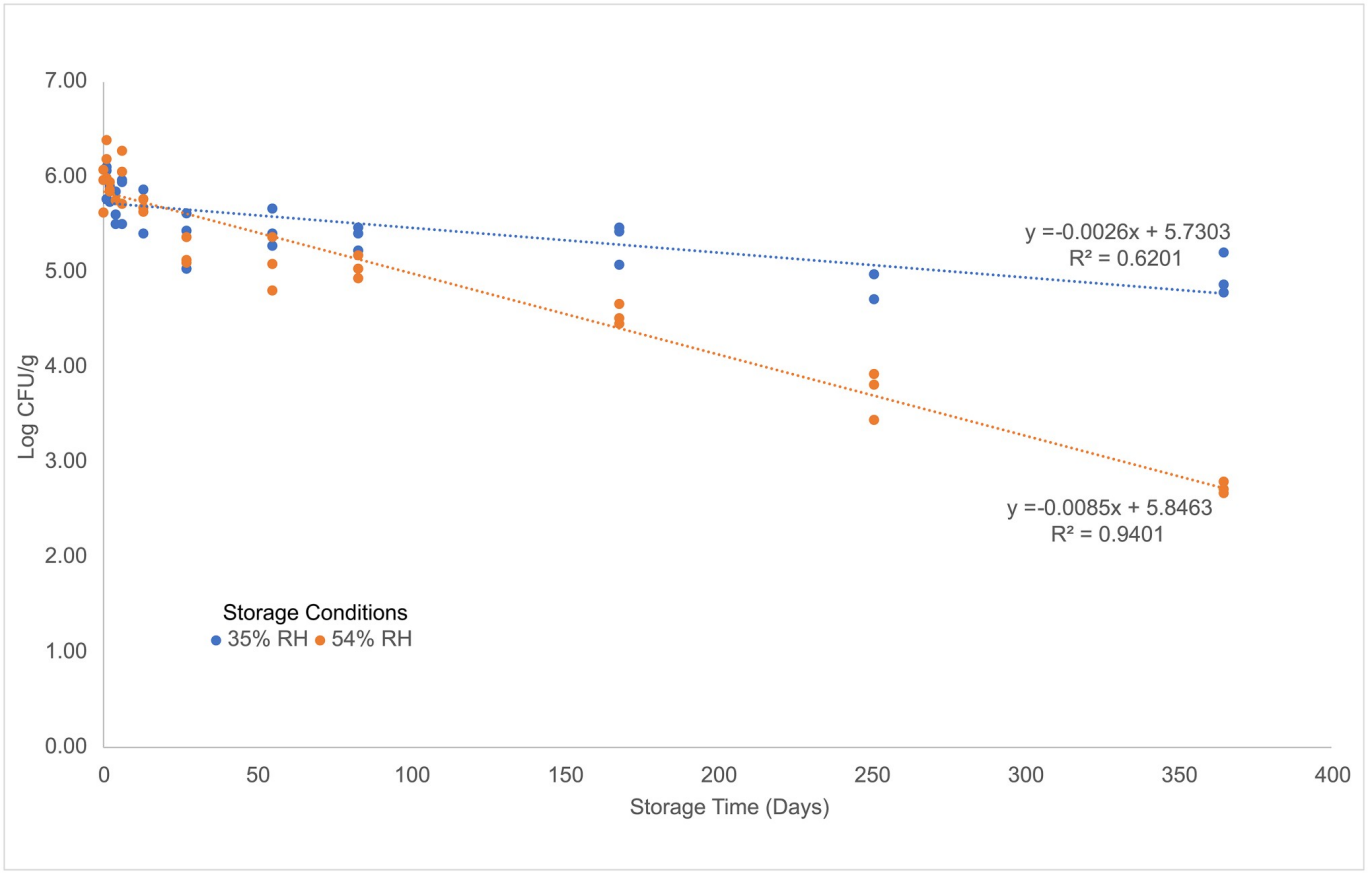
Survival curve of inoculated pistachios with a 5-strain *Salmonella* cocktail stored at different relative humidity levels. Measured *Salmonella* log CFU/g values at different storage time points determined by direct plating methods from pistachios inoculated with a 5-strain cocktail of *Salmonella enterica* stored at 35% RH at 25°C and at 54% RH at 25°C. The regression line for each storage condition is shown as well as the equation and R^2^ value.

*Salmonella* in the five-strain cocktail was able to persist in pistachios for up to one year (the length of the study) (Fig 5). There was a ~1 log CFU/g reduction in *Salmonella* during the period of desiccation (24 hour drying time) prior to storage. The *Salmonella* in the inoculated pistachios stored at 35% relative humidity (mean a_w_ = 0.3398, stdev = 0.0228) were able to persist for a year with only an additional ~1 log CFU/g reduction after desiccation (a total of ~2 log CFU/g reduction). The *Salmonella* in the pistachios stored at 54% relative humidity (mean a_w_ = 0.5337, stdev = 0.0370) showed an additional ~3 log CFU/g reduction over a year. A repeated measures ANOVA analysis showed the impact of humidity condition (high vs low) over time. The rate of reduction was higher for the high humidity condition based on the observed difference in the slope (low and high humidity was -0.0026 and -0.0085 per day, respectively) between the two conditions (*p* < .0001) (storage data shown in S7 Table). Differences in log CFU/g between low and high humidity conditions was most pronounced after 6 months (Fig 6). These findings are consistent with those reported in literature [38, 39, 76].

**Fig 7:**
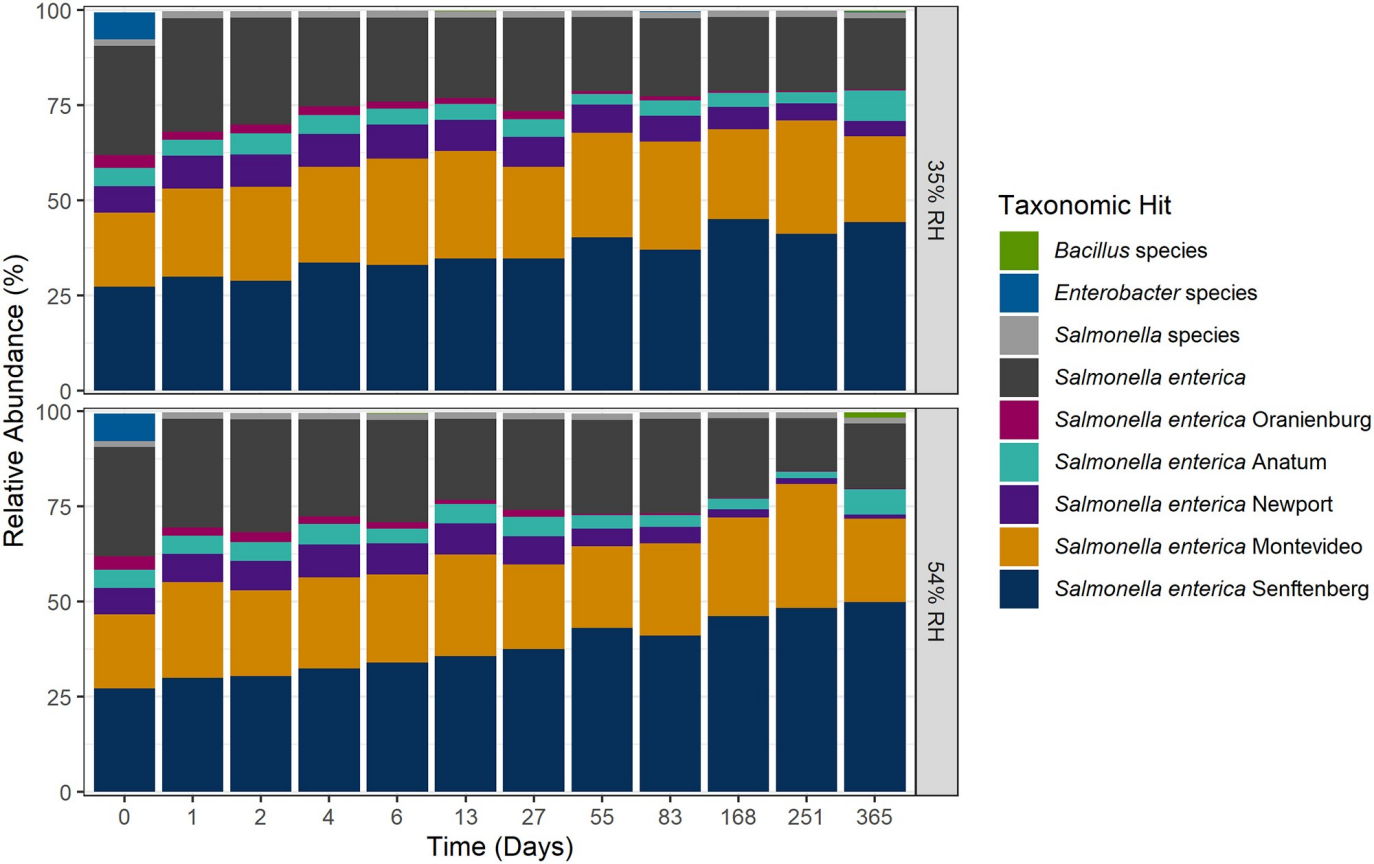
Results from shotgun metagenomic sequencing. Relative abundance of *Salmonella enterica* cocktail and other co-enriching bacterial species for recovery of *Salmonella enterica* from pistachio storage study. Taxonomy was assigned using an in-house developed bacterial kmer database.

One main objective of the study was to determine if there was a difference in which serovars were surviving within the cocktail inoculum. A previous storage study by Kimber et al. [77] evaluated the surviving serovars from the cocktail at the end of the storage study, hence one goal of our study was to analyze the composition of surviving serovars throughout storage, which allows for discrete timepoints of possible serovar elimination. Two methods were used to determine the serovars present, the Luminex xMAP^®^ *Salmonella* serotyping assay and metagenomic shotgun sequencing with data analysis using a custom kmers database. The Luminex assay identified each serovar except for one timepoint at 35% RH and two timepoints at 54% RH (S1 Fig). However, the results from the shotgun metagenomic analysis identified all five serotypes present in each of the triplicate samples at every time point for both storage conditions (Fig 7). Sequencing was conducted post-enrichment, therefore the serovars identified from the raw reads are from viable bacteria. The limitation of the molecular serotyping assay is the number of colonies that are selected and tested. Unintentional bias could be introduced into the results based on colony selection by either over- or under-selection, whereas there is a higher probability of sequencing all strains that are present above a certain threshold with the sequencing method. The Kimber et al. 2012 study used the same Oranienburg strain as in our experiments, but the researchers did not identify this strain in the one hundred colonies they tested at the end of their study (14.5 months) [77]. In contrast, we were able to still recover the Oranienburg strain both in sequencing and by colony picks at 12 months. Two possibilities for the absence of Oranienburg in the Kimber et al. 2012 study could be that Oranienberg was missed due to the bias that can occur when picking colonies for serovar determination or Oranienberg simply did not survive. This could be determined by using metagenomic sequencing (removes colony pick bias) or by picking colonies at different time points (identify survival cutoff). The use of the metagenomic/bioinformatic pathway allows for a robust determination of the cocktail composition while reducing the bias and time of traditional molecular serotyping. Another interesting finding was that the primary source of the *Salmonella* (low-moisture vs. high-moisture environment) may not necessarily determine the ability to survive in a different environment, as shown with the survivability of the Newport strain on the pistachios.

This storage study reinforces the capability for *Salmonella* to survive for extended times on this low-moisture commodity. The results from our study conclude that the five-strain cocktail of *Salmonella* can survive in pistachios and be detected by direct plating methods when stored at different humidity conditions for at least one year. The extended shelf-life of pistachios combined with our evidence of *Salmonella* survival for up to a year highlights the importance of surveillance of this pathogen.

## Conclusions

Pistachios may be contaminated with pathogenic bacteria in the growing, production, and processing environments and present a hazard due to the ability for *Salmonella* to persist in low-moisture environments and foods. During outbreak investigations, epidemiological, traceback, and laboratory information are used to link illnesses to food sources. Diagnostic capabilities have been greatly enhanced due to the development and increasing applicability of WGS, which is now fully deployed as a molecular epidemiological tool to assist in foodborne outbreak investigations [20–22]. We were able to match clinical and food *Salmonella* Senftenberg and Montevideo isolates and define SNP differences between closely related strains by using WGS in two different phylogenetic analyses. Further, by implementing long read sequence technology, we identified the presence of the heavy metal tolerance island (CHASRI) in strains likely associated with preharvest contamination, possibly indicative of their adaptation to the presence of copper in this niche. Presence of the CHASRI in *Salmonella* Senftenberg (ST14) and *Salmonella* Montevideo (ST316) may explain the increased level of recovery of these strains from pistachios or pistachio environments due to selective pressure from copper usage, even though previous data has shown a greater diversity of serovars present [11]. Finally, this analysis points to at least three possible pistachio-associated environments where closely related *Salmonella* Senftenberg and Montevideo strains could have been residing as recently as 2018 including 1) multiple environmental adapted strains in the pre-harvest or orchard environment (ST14 and ST316); 2) a clonal strain at the pistachio handling facilities which could include storage silos (ST185) and 3) a clonal strain in the intermediate post-harvest environment (e.g., shared farm equipment or transport equipment) (ST138).

Our storage study demonstrates that *Salmonella* will survive in pistachios for a minimum of one year at both high and low relative humidity. The use of a non-nut associated strain, *Salmonella* Newport, in our cocktail and its ability to be recovered at the 12-month time point, showed that the original source may not necessarily have a significant impact of survival of *Salmonella* on a low-moisture food. Pistachios may be stored untreated for 12 months or longer by pistachio handlers and then consumers may store them up to an additional 12 months [78]. The ability of *Salmonella* to survive long-term in a dry environment highlights the need for adequate preventive controls in the processing of pistachios. Harris et al. [10] report that majority of the pistachios sold in the U.S. market are roasted inshell. Identification and surveillance of the possible sources of contamination, (e.g., the growing environment, shared equipment, or processing environment) would likely augment implementation of specific production changes that could further reduce the risk of contaminated nut products.

Taken together, this study demonstrates the following: i) evidence of persistent *Salmonella* Senftenberg and Montevideo strains in pistachio environments as recently as 2018, ii) presence of the Copper Homeostasis and Silver Resistance Island (CHASRI) in *Salmonella* Senftenberg and Montevideo strains, suggesting an adaption in response to extrinsic pressures in the pistachio supply chain, and iii) use of metagenomic sequencing in conjunction with storage studies using a cocktail of *Salmonella* strains can identify strains that are surviving over time. This study further emphasizes the power of WGS in conjunction with metadata, which helps to define outbreaks and resident strains and possible routes of contamination, as well as give insights into genetic factors that could lead to fitness in a given niche. Moreover, these findings underscore the importance of industry in adhering to food safety standards established for this commodity.

## Supporting information

S1 Table Isolates and metadata information for our study. (PDF)

S2 Table SNP matrix for ST14 isolates. SNP matrix generated from the CFSAN SNP pipeline for the *Salmonella* Senftenberg isolates from ST14.
(PDF)

S3 Table SNP matrix for ST185 isolates. SNP matrix generated from the CFSAN SNP pipeline for the *Salmonella* Senftenberg isolates from ST185.
(PDF)

S4 Table SNP matrix for ST316 isolates. SNP matrix generated from the CFSAN SNP pipeline for the *Salmonella* Montevideo isolates from ST316.
(PDF)

S5 Table SNP matrix for ST138 isolates. SNP matrix generated from the CFSAN SNP pipeline for the *Salmonella* Montevideo isolates from ST138.
(PDF)

S6 Table Phages identified in study isolates. This table shows the additional phages that were identified by PHASTER in the closed genomes of *Salmonella* Senftenberg and Montevideo from our study.
(PDF)

S7 Table Results of storage study of inoculated pistachios stored at 35% RH and 54% RH sampled at different time points. The direct plating results are shown in this table for triplicate samples taken at different time points throughout the storage study. The water activity measurements taken of these samples is also shown.
(PDF)

S1 Fig Results of the Luminex serotyping of colonies from inoculated pistachios. This figure shows the percentage of serotypes identified at different time points from direct plating of inoculated pistachios stored at 35% RH and 54% RH.
(PDF)

## References

[1] Podolak R, Enache E, Stone W, Black DG, Elliott PH. Sources and risk factors for contamination, survival, persistence, and heat resistance of Salmonella in low-moisture foods. J Food Prot. 2010;73(10):1919–36. doi: 10.4315/0362-028x-73.10.1919 21067682

[2] Beuchat LR, Komitopoulou E, Beckers H, Betts RP, Bourdichon F, Fanning S, et al. Low-water activity foods: increased concern as vehicles of foodborne pathogens. J Food Prot. 2013;76(1):150–72. doi: 10.4315/0362-028X.JFP-12-211 23317872

[3] Brar PK, Danyluk MD. Nuts and Grains: Microbiology and Preharvest Contamination Risks. Microbiol Spectr. 2018;6(2). doi: 10.1128/microbiolspec.PFS-0023-2018 29701166 PMC11633566

[4] Harris LJ, Yada S, Beuchat LR, Danyluk MD. Outbreaks of foodborne illness associated with the consumption of tree nuts, peanuts, and sesame seeds 2019 https://ucfoodsafety.ucdavis.edu/low-moisture-foods/nuts-and-nut-pastes.

[5] Centers for Disease Control and Prevention (CDC). Multistate Outbreak of Salmonella Infections Linked to Pistachio Nuts (FINAL UPDATE) 2009 https://www.cdc.gov/salmonella/2009/pistachio-nuts-4-14-2009.html.

[6] Centers for Disease Control and Prevention (CDC). Multistate outbreak of Salmonella Montevideo and Salmonella Senftenberg infections linked to Wonderful Pistachios (final update): Centers for Disease Control and Prevention, Atlanta, GA.; 2016 https://www.cdc.gov/salmonella/montevideo-03-16/index.html.

[7] Food and Drug Administration (FDA). FDA Investigated Multistate Outbreak of Salmonella Infections Linked to Wonderful Pistachio 2016 https://www.fda.gov/food/outbreaks-foodborne-illness/fda-investigated-multistate-outbreak-salmonella-infections-linked-wonderful-pistachio.

[8] Food and Drug Administration (FDA). FDA Investigation Summary—Multistate Outbreak of Salmonella Senftenberg Infections Associated with Pistachios from a California Roaster 2014 http://outbreakdatabase.com/reports/2013_Salmonella_Senftenberg_Linked_to_Pistachos.pdf.

[9] Yada S, Harris LJ. Recalls of tree nuts and peanuts in the U.S., 2001 to present 2019 09/03/2019. https://ucfoodsafety.ucdavis.edu/low-moisture-foods/nuts-and-nut-pastes.

[10] Food and Drug Administration (FDA). FDA Warns Consumers Not to Eat California Prime Produce and Orange County Orchards Brands of Pistachios Products linked to the previous recall by Setton Pistachio of Terra Bella Inc. 2009 http://wayback.archive-it.org/7993/20170112160300/http://www.fda.gov/NewsEvents/Newsroom/PressAnnouncements/2009/ucm168574.htm.

[11] Harris LJ, Lieberman V, Mashiana RP, Atwill E, Yang M, Chandler JC, et al. Prevalence and Amounts of Salmonella Found on Raw California Inshell Pistachios. J Food Prot. 2016;79(8):1304–15. doi: 10.4315/0362-028X.JFP-16-054 27497117

[12] Uesugi AR, Danyluk MD, Mandrell RE, Harris LJ. Isolation of Salmonella Enteritidis phage type 30 from a single almond orchard over a 5-year period. J Food Prot. 2007;70(8):1784–9. doi: 10.4315/0362-028x-70.8.1784 17803132

[13] California Pistachio Review Board. Good Algricultural Practices Manual Guidelines for California Pistachio Growers 2009. http://calpistachioresearch.org/wp-content/uploads/2017/10/GAP_Manual_2009.pdf.

[14] Theofel CG, Williams TR, Gutierrez E, Davidson GR, Jay-Russell M, Harris LJ. Microorganisms Move a Short Distance into an Almond Orchard from an Adjacent Upwind Poultry Operation. Appl Environ Microbiol. 2020;86(15). 10.1128/AEM.00573-20 PMC7376559 32444472

[15] American Pistachio Growers. Growing and Harvesting American Pistachios 2017 https://americanpistachios.org/growing-and-harvesting.

[16] Agriculture Marketing Resource Center. Pistachios 2018 https://www.agmrc.org/commodities-products/nuts/pistachios.

[17] United States Department of Agriculture. Agricultural Statistics 2019 https://www.nass.usda.gov/Publications/Ag_Statistics/2019/2019_complete_publication.pdf.

[18] Farakos SMS, Pouillot R, Davidson GR, Johnson R, Spungen J, Son I, et al. A Quantitative Risk Assessment of Human Salmonellosis from Consumption of Pistachios in the United States. J Food Prot. 2018;81(6):1001–14. doi: 10.4315/0362-028X.JFP-17-379 29757010

[19] Moussavi M, Lieberman V, Theofel C, Barouei J, Harris LJ. Growth of Salmonella and Other Foodborne Pathogens on Inoculated Inshell Pistachios during Simulated Delays between Hulling and Drying. J Food Prot. 2019;82(5):815–25. doi: 10.4315/0362-028X.JFP-18-450 30995131

[20] Antony L, Behr M, Sockett D, Miskimins D, Aulik N, Christopher-Hennings J, et al. Genome divergence and increased virulence of outbreak associated Salmonella enterica subspecies enterica serovar Heidelberg. Gut Pathog. 2018;10:53. doi: 10.1186/s13099-018-0279-0 30603048 PMC6304783

[21] Hoffmann M, Luo Y, Monday SR, Gonzalez-Escalona N, Ottesen AR, Muruvanda T, et al. Tracing Origins of the Salmonella Bareilly Strain Causing a Food-borne Outbreak in the United States. J Infect Dis. 2016;213(4):502–8. doi: 10.1093/infdis/jiv297 25995194

[22] Wilson MR, Brown E, Keys C, Strain E, Luo Y, Muruvanda T, et al. Whole Genome DNA Sequence Analysis of Salmonella subspecies enterica serotype Tennessee obtained from related peanut butter foodborne outbreaks. PLoS One. 2016;11(6):e0146929. doi: 10.1371/journal.pone.0146929 27258142 PMC4892500

[23] Pightling AW, Pettengill JB, Luo Y, Baugher JD, Rand H, Strain E. Interpreting Whole-Genome Sequence Analyses of Foodborne Bacteria for Regulatory Applications and Outbreak Investigations. Frontiers in microbiology. 2018;9:1482. doi: 10.3389/fmicb.2018.01482 30042741 PMC6048267

[24] Brown E, Dessai U, McGarry S, Gerner-Smidt P. Use of Whole-Genome Sequencing for Food Safety and Public Health in the United States. Foodborne Pathogens and Disease. 2019;16(7):441–50. doi: 10.1089/fpd.2019.2662 31194586 PMC6653787

[25] National Library of Medicine (US) National Center for Biotechnology. The NCBI Pathogen Detection Project https://www.ncbi.nlm.nih.gov/pathogens/.

[26] Allard MW, Strain E, Melka D, Bunning K, Musser SM, Brown EW, et al. Practical Value of Food Pathogen Traceability through Building a Whole-Genome Sequencing Network and Database. J Clin Microbiol. 2016;54(8):1975–83. doi: 10.1128/JCM.00081-16 27008877 PMC4963501

[27] Bankevich A, Nurk S, Antipov D, Gurevich AA, Dvorkin M, Kulikov AS, et al. SPAdes: a new genome assembly algorithm and its applications to single-cell sequencing. J Comput Biol. 2012;19(5):455–77. doi: 10.1089/cmb.2012.0021 22506599 PMC3342519

[28] Alikhan NF, Zhou Z, Sergeant MJ, Achtman M. A genomic overview of the population structure of Salmonella. PLoS Genet. 2018;14(4):e1007261. doi: 10.1371/journal.pgen.1007261 29621240 PMC5886390

[29] Tatusova T, DiCuccio M, Badretdin A, Chetvernin V, Nawrocki EP, Zaslavsky L, et al. NCBI prokaryotic genome annotation pipeline. Nucleic Acids Res. 2016;44(14):6614–24. doi: 10.1093/nar/gkw569 27342282 PMC5001611

[30] Haendiges J, Blessington T, Zheng J, Davidson G, Miller JD, Hoffmann M. Complete Genome Sequences of Four Salmonella enterica subsp. enterica Serovar Senftenberg and Montevideo Isolates Associated with a 2016 Multistate Outbreak in the United States. Genome Announc. 2018;6(26). doi: 10.1128/genomeA.00630-18 29954918 PMC6025934

[31] Haendiges J, Gonzalez-Escalona N, Miller JD, Hoffmann M. Complete Genome Sequences of Four Salmonella enterica Strains Associated with Pistachios Assembled Using a Combination of Short- and Long-Read Sequencing. Microbiol Resour Announc. 2019;8(38). doi: 10.1128/MRA.00975-19 31537673 PMC6753277

[32] Arndt D, Marcu A, Liang Y, Wishart DS. PHAST, PHASTER and PHASTEST: Tools for finding prophage in bacterial genomes. Brief Bioinform. 2017. 10.1093/bib/bbx121 PMC6781593 29028989

[33] Junemann S, Sedlazeck FJ, Prior K, Albersmeier A, John U, Kalinowski J, et al. Updating benchtop sequencing performance comparison. Nat Biotechnol. 2013;31(4):294–6. doi: 10.1038/nbt.2522 23563421

[34] Simon S, Trost E, Bender J, Fuchs S, Malorny B, Rabsch W, et al. Evaluation of WGS based approaches for investigating a food-borne outbreak caused by Salmonella enterica serovar Derby in Germany. Food Microbiol. 2018;71:46–54. doi: 10.1016/j.fm.2017.08.017 29366468

[35] Davis S P J, Luo Y, Payne J, Shpuntoff A, Rand H, Strain E. CFSAN SNP Pipeline: an automated method for constructing SNP matrices from next-generation sequence data. PeerJ. 2015;1(20).

[36] Stamatakis A. RAxML version 8: a tool for phylogenetic analysis and post-analysis of large phylogenies. Bioinformatics. 2014;30(9):1312–3. doi: 10.1093/bioinformatics/btu033 24451623 PMC3998144

[37] Haendiges J, Keller S, Suehr Q, Anderson N, Reed E, Zheng J, et al. Complete Genome Sequences of Five Salmonella enterica Strains Used in Inoculation Cocktails in Low-Moisture Food Storage Studies. Microbiol Resour Announc. 2019;8(2). 10.1128/MRA.01588-18 PMC6328678 30643905

[38] Keller SE, VanDoren JM, Grasso EM, Halik LA. Growth and survival of Salmonella in ground black pepper (Piper nigrum). Food Microbiol. 2013;34(1):182–8. doi: 10.1016/j.fm.2012.12.002 23498196

[39] Farakos SMS, Pouillot R, Keller SE. Salmonella Survival Kinetics on Pecans, Hazelnuts, and Pine Nuts at Various Water Activities and Temperatures. J Food Prot. 2017;80(5):879–85. doi: 10.4315/0362-028X.JFP-16-392 28414256

[40] McQuiston JR, Waters RJ, Dinsmore BA, Mikoleit ML, Fields PI. Molecular determination of H antigens of Salmonella by use of a microsphere-based liquid array. J Clin Microbiol. 2011;49(2):565–73. doi: 10.1128/JCM.01323-10 21159932 PMC3043481

[41] Fitzgerald C, Collins M, van Duyne S, Mikoleit M, Brown T, Fields P. Multiplex, bead-based suspension array for molecular determination of common Salmonella serogroups. J Clin Microbiol. 2007;45(10):3323–34. doi: 10.1128/JCM.00025-07 17634307 PMC2045348

[42] Patro JN, Ramachandran P, Barnaba T, Mammel MK, Lewis JL, Elkins CA. Culture-Independent Metagenomic Surveillance of Commercially Available Probiotics with High-Throughput Next-Generation Sequencing. mSphere. 2016;1(2). doi: 10.1128/mSphere.00057-16 27303722 PMC4894680

[43] Allard MW, Luo Y, Strain E, Li C, Keys CE, Son I, et al. High resolution clustering of Salmonella enterica serovar Montevideo strains using a next-generation sequencing approach. BMC Genomics. 2012;13:32. doi: 10.1186/1471-2164-13-32 22260654 PMC3368722

[44] Staehlin BM, Gibbons JG, Rokas A, O’Halloran TV, Slot JC. Evolution of a Heavy Metal Homeostasis/Resistance Island Reflects Increasing Copper Stress in Enterobacteria. Genome Biol Evol. 2016;8(3):811–26. doi: 10.1093/gbe/evw031 26893455 PMC4824010

[45] Johnson M, Zaretskaya I, Raytselis Y, Merezhuk Y, McGinnis S, Madden TL. NCBI BLAST: a better web interface. Nucleic Acids Res. 2008;36(Web Server issue):W5–9. doi: 10.1093/nar/gkn201 18440982 PMC2447716

[46] Arai N, Sekizuka T, Tamamura Y, Kusumoto M, Hinenoya A, Yamasaki S, et al. Salmonella Genomic Island 3 Is an Integrative and Conjugative Element and Contributes to Copper and Arsenic Tolerance of Salmonella enterica. Antimicrob Agents Chemother. 2019;63(9). 10.1128/AAC.00429-19 PMC6709492 31209002

[47] Mourao J, Rebelo A, Ribeiro S, Peixe L, Novais C, Antunes P. Tolerance to arsenic contaminant among multidrug-resistant and copper-tolerant Salmonella successful clones is associated with diverse ars operons and genetic contexts. Environ Microbiol. 2020. doi: 10.1111/1462-2920.15016 32281716

[48] Campos J, Cristino L, Peixe L, Antunes P. MCR-1 in multidrug-resistant and copper-tolerant clinically relevant Salmonella 1,4,[5],12:i:- and S. Rissen clones in Portugal, 2011 to 2015. Euro Surveill. 2016;21(26). 10.2807/1560-7917.ES.2016.21.26.30270 27387036

[49] Adaskaveg JE, Gubler D, Michailides T. Efficacy and Timing of Fungicides, Bactericides, and Biologicals for Deciduous Tree Fruit, Nut, Strawberry, and Vine Crops 2017 https://www2.ipm.ucanr.edu/agriculture/pistachio.

[50] Sym-Agro;. California Tree Nut Growers Now Have Systemic Copper in Their Toolboxes with Instill bactericide and fungicide 2019 https://sym-agro.com/california-tree-nut-growers-now-have-systemic-copper-in-their-toolboxes/.

[51] Beede RH, Brown PH, Kallsen C, Weinbaum SA. Diagnosing and correcting nutrient deficiences. In: Ferguson L, Haviland DR, editors. Pistachio Production Manual. Division of Agriculture and Natural Resources, University of California, Oakland 2016. doi: 10.1152/ajpregu.00528.2015

[52] Beede RH. Pistachio Micronutrient Management 2017 https://ucanr.edu/sites/PistachioShortCourse/files/274450.pdf.

[53] Carroll LM, Gaballa A, Guldimann C, Sullivan G, Henderson LO, Wiedmann M. Identification of Novel Mobilized Colistin Resistance Gene mcr-9 in a Multidrug-Resistant, Colistin-Susceptible Salmonella enterica Serotype Typhimurium Isolate. MBio. 2019;10(3). 10.1128/mBio.00853-19 PMC6509194 31064835

[54] Kieffer N, Royer G, Decousser JW, Bourrel AS, Palmieri M, Ortiz De La Rosa JM, et al. mcr-9, an Inducible Gene Encoding an Acquired Phosphoethanolamine Transferase in Escherichia coli, and Its Origin. Antimicrob Agents Chemother. 2019;63(9). 10.1128/AAC.00965-19 PMC6709461 31209009

[55] Wahl A, Battesti A, Ansaldi M. Prophages in Salmonella enterica: a driving force in reshaping the genome and physiology of their bacterial host? Mol Microbiol. 2019;111(2):303–16. doi: 10.1111/mmi.14167 30466179 PMC7380047

[56] Pearce ME, Alikhan NF, Dallman TJ, Zhou Z, Grant K, Maiden MCJ. Comparative analysis of core genome MLST and SNP typing within a European Salmonella serovar Enteritidis outbreak. Int J Food Microbiol. 2018;274:1–11. doi: 10.1016/j.ijfoodmicro.2018.02.023 29574242 PMC5899760

[57] Wang YU, Pettengill JB, Pightling A, Timme R, Allard M, Strain E, et al. Genetic Diversity of Salmonella and Listeria Isolates from Food Facilities. J Food Prot. 2018;81(12):2082–9. doi: 10.4315/0362-028X.JFP-18-093 30485763

[58] Wuyts V, Denayer S, Roosens NH, Mattheus W, Bertrand S, Marchal K, et al. Whole Genome Sequence Analysis of Salmonella Enteritidis PT4 Outbreaks from a National Reference Laboratory’s Viewpoint. PLoS Curr. 2015;7. doi: 10.1371/currents.outbreaks.aa5372d90826e6cb0136ff66bb7a62fc 26468422 PMC4593640

[59] Taylor AJ, Lappi V, Wolfgang WJ, Lapierre P, Palumbo MJ, Medus C, et al. Characterization of Foodborne Outbreaks of Salmonella enterica Serovar Enteritidis with Whole-Genome Sequencing Single Nucleotide Polymorphism-Based Analysis for Surveillance and Outbreak Detection. J Clin Microbiol. 2015;53(10):3334–40. doi: 10.1128/JCM.01280-15 26269623 PMC4572550

[60] Octavia S, Wang Q, Tanaka MM, Kaur S, Sintchenko V, Lan R. Delineating community outbreaks of Salmonella enterica serovar Typhimurium by use of whole-genome sequencing: insights into genomic variability within an outbreak. J Clin Microbiol. 2015;53(4):1063–71. doi: 10.1128/JCM.03235-14 25609719 PMC4365247

[61] Leekitcharoenphon P, Nielsen EM, Kaas RS, Lund O, Aarestrup FM. Evaluation of whole genome sequencing for outbreak detection of Salmonella enterica. PLoS One. 2014;9(2):e87991. doi: 10.1371/journal.pone.0087991 24505344 PMC3913712

[62] Esmaeili A, Khoram MR, Gholami M, Eslami H. Pistachio waste management using combined composting-vermicomposting technique: Physico-chemical changes and worm growth analysis. Journal of Cleaner Production. 2020;242.

[63] Jalili M, Mokhtari M, Eslami H, Abbasi F, Ghanbari R, Ebrahimi AA. Toxicity evaluation and management of co-composting pistachio wastes combined with cattle manure and municipal sewage sludge. Ecotoxicol Environ Saf. 2019;171:798–804. doi: 10.1016/j.ecoenv.2019.01.056 30660973

[64] Karagoktas M, Uygur V, Aydogdu M, Rastgeldi I. The effects of pistachio nut hull compost on soil fertility: A comparative study with manure. 9th International Soil Science Congress; Turkey2014.

[65] Andrews EM, Kassama S, Smith EE, Brown PH, Khalsa SDS. A Review of Potassium-Rich Crop Residues Used as Organic Matter Amendments in Tree Crop Agroecosystems. Agriculture. 2021;11(7):580.

[66] Fernandez-Bayo JD, Shea EA, Parr AE, Achmon Y, Stapleton JJ, VanderGheynst JS, et al. Almond processing residues as a source of organic acid biopesticides during biosolarization. Waste Manag. 2020;101:74–82. doi: 10.1016/j.wasman.2019.09.028 31604160 PMC8759351

[67] Deng X, Desai PT, den Bakker HC, Mikoleit M, Tolar B, Trees E, et al. Genomic epidemiology of Salmonella enterica serotype Enteritidis based on population structure of prevalent lineages. Emerg Infect Dis. 2014;20(9):1481–9. doi: 10.3201/eid2009.131095 25147968 PMC4178404

[68] Hoffmann M, Miller J, Melka D, Allard MW, Brown EW, Pettengill JB. Temporal Dynamics of Salmonella enterica subsp. enterica Serovar Agona Isolates From a Recurrent Multistate Outbreak. Front Microbiol. 2020;11:478. doi: 10.3389/fmicb.2020.00478 32265893 PMC7104706

[69] Pettengill JB, Markell A, Conrad A, Carleton H, Beal J, Rand H, et al. A multinational listeriosis outbreak and the importance of sharing genomic data. Lancet Microbe. 2020;1(6):E233–4. 10.1016/S2666-5247(20)30122-1 PMC10966468 35544215

[70] Villaverde C, Baucells MD, Manzanilla EG, Barroeta AC. High levels of dietary unsaturated fat decrease alpha-tocopherol content of whole body, liver, and plasma of chickens without variations in intestinal apparent absorption. Poult Sci. 2008;87(3):497–505. doi: 10.3382/ps.2007-00292 18281576

[71] Wang J, Singh AK, Kong F, Kim WK. Effect of almond hull as an alternative ingredient on broiler performance, nutrient digestibility, and cecal microbiota diversity. Poultry Science. 2021;100(3). 10.1016/j.psj.2020.11.030 PMC7936146 33516473

[72] Akande TO, Akinwumi AO, Abegunde TO. Cashew reject meal in diets of laying chickens: nutritional and economic suitability. J Anim Sci Technol. 2015;57:17. doi: 10.1186/s40781-015-0051-7 26290737 PMC4540253

[73] Setton Farms. Sustainablity 2013 www.settonfarms.com/inside-setton/sustainability.

[74] Touchstone Pistachio Co. Sustainability 2020 www.touchstonepistachio.com/sustainability.

[75] Rock M, Buntain BJ, Hatfield JM, Hallgrimsson B. Animal-human connections, “one health,” and the syndemic approach to prevention. Soc Sci Med. 2009;68(6):991–5. doi: 10.1016/j.socscimed.2008.12.047 19157669

[76] Santillana Farakos SM, Pouillot R, Anderson N, Johnson R, Son I, Van Doren J. Modeling the survival kinetics of Salmonella in tree nuts for use in risk assessment. Int J Food Microbiol. 2016;227:41–50. doi: 10.1016/j.ijfoodmicro.2016.03.014 27062527

[77] Kimber MA, Kaur H, Wang L, Danyluk MD, Harris LJ. Survival of Salmonella, Escherichia coli O157:H7, and Listeria monocytogenes on inoculated almonds and pistachios stored at -19, 4, and 24 degrees C. J Food Prot. 2012;75(8):1394–403. doi: 10.4315/0362-028X.JFP-12-023 22856562

[78] Lee LE, Metz D, Giovanni M, Bruhn CM. Consumer knowledge and handling of tree nuts: food safety implications. Food Protection Trends. 2011(31):18–27.

