# Supplementary material for: Genomic evidence of environmental and resident Salmonella Senftenberg and Montevideo contamination in the pistachio supply-chain
Source: PLoS One. 2021 Nov 4;16(11):e0259471. doi: 10.1371/journal.pone.0259471 (PMC8568146; doi:10.1371/journal.pone.0259471)
Supplement: S1 Table — (PDF) [file pone.0259471.s001.pdf]

## S1 Table: Isolates and Metadata information for our study

| Strain | Location | Isolation Source | SNP cluster | ST | Serovar | SRA | Collection Date | Facility |
|---|---|---|---|---|---|---|---|---|
| 2016K-0180 | USA | Clinical | PDS000031814 | 14 | Senftenberg | SRR3315923 | Missing | |
| 2017K-0050 | USA | Clinical | PDS000031814 | 14 | Senftenberg | SRR5238274 | 2016 | |
| CFSAN000622 | USA:MD | Chicken | PDS000031814 | 14 | Senftenberg | SRR1060630 | 1987 | |
| CFSAN015119 | USA | Pistachios | PDS000031814 | 14 | Senftenberg | SRR2585751 | 2009 | |
| CFSAN015122 | USA | Shelled Pistachios | PDS000031814 | 14 | Senftenberg | SRR2585428 | 2009 | |
| CFSAN016599 | USA | Pistachios | PDS000031814 | 14 | Senftenberg | SRR1816865 | 2009 | B |
| CFSAN045763 | USA:CA | Raw Pistachios | PDS000031814 | 14 | Senftenberg | SRR3168967 | 2016 | A |
| CFSAN047567 | USA:CA | Pistachios | PDS000031814 | 14 | Senftenberg | SRR3309442 | 2016 | A |
| CFSAN047866 | USA:TX | Pistachios | PDS000031814 | 14 | Senftenberg | SRR3272258 | 2016 | A |
| CFSAN048426 | USA:CA | Raw Pistachio | PDS000031814 | 14 | Senftenberg | SRR3340981 | 2016 | A |
| CFSAN048427 | USA:CA | Raw Pistachio | PDS000031814 | 14 | Senftenberg | SRR3340982 | 2016 | A |
| CFSAN048428 | USA:CA | Raw Pistachio | PDS000031814 | 14 | Senftenberg | SRR3340985 | 2016 | A |
| CFSAN048429 | USA:CA | Raw Pistachio | PDS000031814 | 14 | Senftenberg | SRR3340986 | 2016 | A |
| CFSAN058295 | USA: TX | Shelled Pistachios | PDS000031814 | 14 | Senftenberg | SRR5120752 | 2016 | A |
| FMA0102 | Canada | Pistachios | PDS000031814 | 14 | Senftenberg | SRR1258573 | 2013 | |
| OSF033320 | USA:CA | Pistachios | PDS000031814 | 14 | Senftenberg | SRR2890018 | 2014 | |
| OSF033321 | USA:CA | Pistachios | PDS000031814 | 14 | Senftenberg | SRR2890019 | 2014 | |
| OSF047785 | USA: TX | Pistachios | PDS000031814 | 14 | Senftenberg | SRR3286882 | 2015 | |
| OSF048599 | USA:CA | Pistachios | PDS000031814 | 14 | Senftenberg | SRR3372311 | 2016 | |
| OSF048657 | USA:CA | Pistachios | PDS000031814 | 14 | Senftenberg | SRR3391910 | 2016 | |
| OSF048658 | USA:CA | Pistachios | PDS000031814 | 14 | Senftenberg | SRR3391911 | 2016 | |
| OSF048659 | USA:CA | Pistachios | PDS000031814 | 14 | Senftenberg | SRR3391913 | 2016 | |
| OSF048660 | USA:CA | Pistachios | PDS000031814 | 14 | Senftenberg | SRR3657446 | 2016 | |
| OSF048786 | USA:CA | Pistachios | PDS000031814 | 14 | Senftenberg | SRR3437480 | 2016 | |
| OSF049331 | USA:CA | Pistachios | PDS000031814 | 14 | Senftenberg | SRR3490039 | 2016 | |
| OSF049332 | USA:CA | Pistachios | PDS000031814 | 14 | Senftenberg | SRR3490040 | 2016 | |
| OSF056360 | USA:CA | Pistachios | PDS000031814 | 14 | Senftenberg | SRR5057162 | 2016 | |
| OSF056361 | USA:CA | Pistachios | PDS000031814 | 14 | Senftenberg | SRR5057161 | 2016 | |
| OSF056362 | USA:CA | Pistachios | PDS000031814 | 14 | Senftenberg | SRR5057160 | 2016 | |
| OSF056593 | USA:CA | Pistachios | PDS000031814 | 14 | Senftenberg | SRR5070635 | 2014 | |
| OSF056809 | USA:CA | Raw Almonds/nuts | PDS000031814 | 14 | Senftenberg | SRR5116313 | 2014 | F |
| OSF056838 | USA:CA | Raw Pistachios | PDS000031814 | 14 | Senftenberg | SRR5120068 | 2014 | |
| OSF058109 | USA:WA | Raw Pistachios | PDS000031814 | 14 | Senftenberg | SRR5152278 | 2014 | |
| OSF059119 | USA:WA | Pistachios | PDS000031814 | 14 | Senftenberg | SRR5182252 | 2016 | |
| OSF059213 | USA:CA | Environmental Sponge | PDS000031814 | 14 | Senftenberg | SRR5217564 | 2016 | G |
| OSF059253 | USA:CA | Pistachios | PDS000031814 | 14 | Senftenberg | SRR5217444 | 2016 | |
| OSF059265 | USA:CA | Pistachios | PDS000031814 | 14 | Senftenberg | SRR5217482 | 2016 | |
| OSF059289 | USA:WA | Pistachios | PDS000031814 | 14 | Senftenberg | SRR5229127 | 2016 | |
| OSF059786 | USA:CA | Pistachios | PDS000031814 | 14 | Senftenberg | SRR5279048 | 2016 | |

| | | | | | | | | |
|---|---|---|---|---|---|---|---|---|
| OSF059815 | USA:CA | Pistachios | PDS000031814 | 14 | Senftenberg | SRR5251448 | 2016 | |
| OSF067866 | USA:CA | Fertilizer, Bone Meal | PDS000031814 | 14 | Senftenberg | SRR5990714 | 2015 | |
| OSF070289 | USA:CA | Environmental Sponge | PDS000031814 | 14 | Senftenberg | SRR6179039 | 2017 | |
| OSF073486 | USA:CA | Environmental Sponge | PDS000031814 | 14 | Senftenberg | SRR6386672 | 2016 | |
| OSF073577 | USA:CA | Pistachios | PDS000031814 | 14 | Senftenberg | SRR6424913 | 2016 | |
| OSF074224 | USA:CA | Pistachios | PDS000031814 | 14 | Senftenberg | SRR6487131 | 2016 | |
| OSF084195 | USA:CA | Environmental Swab | PDS000031814 | 14 | Senftenberg | SRR7643277 | 2017 | E |
| OSF090574 | USA:TX | Shelled Pistachios | PDS000031814 | 14 | Senftenberg | SRR8428217 | 2016 | |
| OSF090575 | USA:TX | Shelled Pistachios | PDS000031814 | 14 | Senftenberg | SRR8428324 | 2016 | |
| PNUSAS001664 | USA | Clinical | PDS000031814 | 14 | Senftenberg | SRR3240307 | 2016 | |
| PNUSAS001739 | USA | Clinical | PDS000031814 | 14 | Senftenberg | SRR3289842 | 2016 | |
| PNUSAS001800 | USA | Clinical | PDS000031814 | 14 | Senftenberg | SRR3392769 | Missing | |
| PNUSAS008582 | USA | Clinical | PDS000031814 | 14 | Senftenberg | SRR5260040 | 2015 | |
| PNUSAS140610 | USA | Clinical | PDS000031814 | 14 | Senftenberg | SRR11462365 | Missing | |
| SGSC 2516 | USA:MD | Chicken | PDS000031814 | 14 | Senftenberg | SRR5598938 | 1987 | |
| CFSAN017737 | USA:CA | Environmental Swab | PDS000031739 | 185 | Senftenberg | SRR3151938 | 2013 | C |
| CFSAN017738 | USA:CA | Environmental Swab | PDS000031739 | 185 | Senftenberg | SRR3151939 | 2013 | C |
| CFSAN017739 | USA:CA | Environmental Swab | PDS000031739 | 185 | Senftenberg | SRR3151940 | 2013 | C |
| CFSAN017740 | USA:CA | Environmental Swab | PDS000031739 | 185 | Senftenberg | SRR3151941 | 2013 | C |
| CFSAN017741 | USA:CA | Environmental Swab | PDS000031739 | 185 | Senftenberg | SRR3151942 | 2013 | C |
| CFSAN017744 | USA:CA | Environmental Swab | PDS000031739 | 185 | Senftenberg | SRR3151943 | 2013 | C |
| CFSAN017745 | USA:CA | Environmental Swab | PDS000031739 | 185 | Senftenberg | SRR3151944 | 2013 | C |
| CFSAN017746 | USA:CA | Environmental Swab | PDS000031739 | 185 | Senftenberg | SRR3242178 | 2013 | C |
| CFSAN017747 | USA:CA | Environmental Swab | PDS000031739 | 185 | Senftenberg | SRR3242179 | 2013 | C |
| CFSAN017748 | USA:CA | Environmental Swab | PDS000031739 | 185 | Senftenberg | SRR3242180 | 2013 | C |
| CFSAN017749 | USA:CA | Environmental Swab | PDS000031739 | 185 | Senftenberg | SRR3242181 | 2013 | C |
| CFSAN017750 | USA:CA | Environmental Swab | PDS000031739 | 185 | Senftenberg | SRR3242182 | 2013 | C |
| CFSAN017751 | USA:CA | Environmental Swab | PDS000031739 | 185 | Senftenberg | SRR3242184 | 2013 | C |
| CFSAN017752 | USA:CA | Environmental Swab | PDS000031739 | 185 | Senftenberg | SRR3242185 | 2013 | C |
| CFSAN017753 | USA:CA | Environmental Swab | PDS000031739 | 185 | Senftenberg | SRR3340442 | 2013 | C |
| CFSAN017754 | USA:CA | Environmental Swab | PDS000031739 | 185 | Senftenberg | SRR3340443 | 2013 | C |
| CFSAN032229 | USA:CA | Environmental Swab | PDS000031739 | 185 | Senftenberg | SRR1979272 | 2015 | C |
| CFSAN032230 | USA:CA | Environmental Swab | PDS000031739 | 185 | Senftenberg | SRR1982146 | 2015 | C |
| CFSAN032231 | USA:CA | Environmental Swab | PDS000031739 | 185 | Senftenberg | SRR1979316 | 2015 | C |
| CFSAN086744 | USA:PA | Pistachios | PDS000031739 | 185 | Senftenberg | SRR8113236 | 2018 | C |
| CFSAN087304 | USA:NJ | Pistachios | PDS000031739 | 185 | Senftenberg | SRR8261878 | 2018 | C |
| FNW19i50 | USA:CA | Pistachios | PDS000031739 | 185 | Senftenberg | SRR1614998 | 2013 | C |

| | | | | | | | | |
|---|---|---|---|---|---|---|---|---|
| FNW19i51 | USA:CA | Pistachios | PDS000031739 | 185 | Senftenberg | SRR1646543 | 2013 | C |
| FSE0085 | Mexico | Tahini | PDS000031739 | 185 | Senftenberg | SRR1212317 | 2011 | |
| FSF0102 | USA:CA | Environmental Swab | PDS000031739 | 185 | Senftenberg | SRR1535718 | 2013 | C |
| FSW0103 | USA:CA | Dry Roasted Pistachios | PDS000031739 | 185 | Senftenberg | SRR1257291 | 2013 | C* |
| FSW0104 | USA:CA | Dry Roasted Pistachios | PDS000031739 | 185 | Senftenberg | SRR1257282 | 2013 | C* |
| OSF056673 | USA:CA | Environmental Swab | PDS000031739 | 185 | Senftenberg | SRR5099892 | 2014 | C |
| OSF056674 | USA:CA | Environmental Swab | PDS000031739 | 185 | Senftenberg | SRR5099891 | 2014 | C |
| OSF056675 | USA:CA | Environmental Swab | PDS000031739 | 185 | Senftenberg | SRR5099888 | 2014 | C |
| OSF056676 | USA:CA | Environmental Swab | PDS000031739 | 185 | Senftenberg | SRR5099890 | 2014 | C |
| OSF056753 | USA:CA | Environmental Swab | PDS000031739 | 185 | Senftenberg | SRR5105418 | 2014 | C |
| OSF056767 | USA:CA | Environmental Swab | PDS000031739 | 185 | Senftenberg | SRR5116302 | 2014 | C |
| OSF056768 | USA:CA | Environmental Swab | PDS000031739 | 185 | Senftenberg | SRR5116309 | 2014 | C |
| OSF056769 | USA:CA | Environmental Swab | PDS000031739 | 185 | Senftenberg | SRR5116457 | 2014 | C |
| OSF057938 | USA:CA | Environmental Swab | PDS000031739 | 185 | Senftenberg | SRR5120074 | 2014 | D |
| OSF057939 | USA:CA | Environmental Swab | PDS000031739 | 185 | Senftenberg | SRR5120108 | 2014 | D |
| OSF057940 | USA:CA | Environmental Swab | PDS000031739 | 185 | Senftenberg | SRR5120103 | 2014 | D |
| OSF057941 | USA:CA | Environmental Swab | PDS000031739 | 185 | Senftenberg | SRR5120104 | 2014 | D |
| OSF057942 | USA:CA | Environmental Swab | PDS000031739 | 185 | Senftenberg | SRR5120105 | 2014 | D |
| OSF057943 | USA:CA | Environmental Swab | PDS000031739 | 185 | Senftenberg | SRR5120107 | 2014 | D |
| OSF057945 | USA:CA | Environmental Swab | PDS000031739 | 185 | Senftenberg | SRR5120102 | 2014 | D |
| OSF057946 | USA:CA | Environmental Swab | PDS000031739 | 185 | Senftenberg | SRR5230332 | 2014 | D |
| OSF057947 | USA:CA | Environmental Swab | PDS000031739 | 185 | Senftenberg | SRR5230170 | 2014 | D |
| OSF057948 | USA:CA | Environmental Swab | PDS000031739 | 185 | Senftenberg | SRR5230325 | 2014 | D |
| OSF057951 | USA:CA | Environmental Swab | PDS000031739 | 185 | Senftenberg | SRR5230171 | 2014 | D |
| OSF057978 | USA:CA | Raw Pistachios | PDS000031739 | 185 | Senftenberg | SRR5230465 | 2014 | C |
| OSF057979 | USA:CA | Raw Pistachios | PDS000031739 | 185 | Senftenberg | SRR5230320 | 2014 | C |
| OSF057980 | USA:CA | Raw Pistachios | PDS000031739 | 185 | Senftenberg | SRR5230330 | 2014 | C |
| OSF057981 | USA:CA | Raw Pistachios | PDS000031739 | 185 | Senftenberg | SRR5230168 | 2014 | C |
| OSF057982 | USA:CA | Raw Pistachios | PDS000031739 | 185 | Senftenberg | SRR5230385 | 2014 | C |
| OSF087751 | USA:CA | Finished Product Pistachios | PDS000031739 | 185 | Senftenberg | SRR8176592 | 2018 | C |
| 2013K-0731 | USA | Clinical | PDS000031739 | 185 | Senftenberg | SRR14760254 | | |
| 2013K-0735 | USA | Clinical | PDS000031739 | 185 | Senftenberg | SRR14760897 | | |
| 2013K-0777 | USA | Clinical | PDS000031739 | 185 | Senftenberg | SRR14766322 | | |
| 44713 | United Kingdom | Clinical | PDS000027237 | 316 | Montevideo | SRR1957791 | 2014 | |
| 378603 | United Kingdom | Clinical | PDS000027237 | 316 | Montevideo | SRR8568765 | 2017 | |
| 533421 | United Kingdom | Clinical | PDS000027237 | 316 | Montevideo | SRR8515722 | 2018 | |

| | | | | | | | | |
|---|---|---|---|---|---|---|---|---|
| 570824 | United Kingdom | Clinical | PDS000027237 | 316 | Montevideo | SRR8484198 | 2018 | |
| 708223 | United Kingdom | Clinical | PDS000027237 | 316 | Montevideo | SRR8774349 | 2019 | |
| 315731156 | Missing | Pistachios | PDS000027237 | 316 | Montevideo | SRR500745 | 2009 | B |
| 2016K-0167 | USA | Clinical | PDS000027237 | 316 | Montevideo | SRR3278065 | Missing | |
| CFSAN000244 | Missing | Pistachios | PDS000027237 | 316 | Montevideo | SRR500472 | 2009 | B |
| CFSAN013604 | USA | Pistachios | PDS000027237 | 316 | Montevideo | SRR2559381 | 2008 | |
| CFSAN014882 | USA | Pistachios | PDS000027237 | 316 | Montevideo | SRR1973753 | 2009 | |
| CFSAN015113 | USA | Blend Trail Mix | PDS000027237 | 316 | Montevideo | SRR2559383 | 2008 | B |
| CFSAN015114 | USA | Blend Trail Mix | PDS000027237 | 316 | Montevideo | SRR2586832 | 2008 | B |
| CFSAN015115 | USA | Pistachios | PDS000027237 | 316 | Montevideo | SRR2585772 | 2009 | B |
| CFSAN015116 | USA | Blend Trail Mix | PDS000027237 | 316 | Montevideo | SRR2586844 | 2009 | B |
| CFSAN015117 | USA | cashew/almond/ pistachio blend | PDS000027237 | 316 | Montevideo | SRR2585430 | 2009 | B |
| CFSAN015118 | USA | Pistachios | PDS000027237 | 316 | Montevideo | SRR2585429 | 2009 | B |
| CFSAN015120 | USA | Pistachios | PDS000027237 | 316 | Montevideo | SRR2585750 | 2009 | B |
| CFSAN015123 | USA | Blend Trail Mix | PDS000027237 | 316 | Montevideo | SRR2559382 | 2008 | B |
| CFSAN016379 | USA:CA | Environmental Swab | PDS000027237 | 316 | Montevideo | SRR1816812 | 2009 | B |
| CFSAN016380 | USA:CA | roasted & salted garlic/onion pistachios | PDS000027237 | 316 | Montevideo | SRR1816860 | 2009 | B |
| CFSAN016411 | USA:CA | Environmental Swab | PDS000027237 | 316 | Montevideo | SRR1816817 | 2009 | B |
| CFSAN016412 | USA:CA | Environmental Swab | PDS000027237 | 316 | Montevideo | SRR1816831 | 2009 | B |
| CFSAN016600 | USA | Pistachios | PDS000027237 | 316 | Montevideo | SRR1816838 | 2009 | B |
| CFSAN016601 | USA | Pistachios | PDS000027237 | 316 | Montevideo | SRR1816839 | 2009 | B |
| CFSAN037640 | USA:IL | Shell Pistachios | PDS000027237 | 316 | Montevideo | SRR3038647 | 2015 | A |
| CFSAN045764 | USA:CA | Raw Pistachios | PDS000027237 | 316 | Montevideo | SRR3168966 | 2016 | A |
| CFSAN047166 | USA:CA | Pistachios | PDS000027237 | 316 | Montevideo | SRR3242198 | 2016 | A |
| CFSAN047167 | USA:CA | Pistachios | PDS000027237 | 316 | Montevideo | SRR3242197 | 2016 | A |
| CFSAN047168 | USA:CA | Pistachios | PDS000027237 | 316 | Montevideo | SRR3242196 | 2016 | A |
| CFSAN047169 | USA:CA | Pistachios | PDS000027237 | 316 | Montevideo | SRR3242195 | 2016 | A |
| CFSAN051296 | USA | Raw Pistachios | PDS000027237 | 316 | Montevideo | SRR3707433 | 2016 | |
| CFSAN064799 | USA | Shelled Pistachios | PDS000027237 | 316 | Montevideo | SRR5758423 | 2017 | K |
| FSIS1606641 | USA:MI | Swine | PDS000027237 | 316 | Montevideo | SRR3555186 | 2016 | |
| FSIS1709808 | USA:GA | Raw Intact Chicken | PDS000027237 | 316 | Montevideo | SRR5196071 | 2016 | |
| FSL_R8-4916 | Missing | Missing | PDS000027237 | 316 | Montevideo | SRR493332 | Missing | |
| FSL_R8-4922 | Missing | Missing | PDS000027237 | 316 | Montevideo | SRR493336 | Missing | |
| OSF045712 | USA:IN | Chicken | PDS000027237 | 316 | Montevideo | SRR3191464 | 2015 | H |
| OSF046987 | USA:CA | Nuts | PDS000027237 | 316 | Montevideo | SRR3225384 | 2015 | F |
| OSF047721 | USA:CA | Pistachios | PDS000027237 | 316 | Montevideo | SRR3294561 | 2015 | |
| OSF051202 | USA:WA | Pistachios | PDS000027237 | 316 | Montevideo | SRR3606932 | 2016 | |
| OSF056837 | USA:CA | Raw Pistachios | PDS000027237 | 316 | Montevideo | SRR5120069 | 2014 | |
| OSF059120 | USA:WA | Pistachios | PDS000027237 | 316 | Montevideo | SRR5182248 | 2016 | |
| OSF069055 | USA:CA | Environmental sponge | PDS000027237 | 316 | Montevideo | SRR6108679 | 2015 | |

| | | | | | | | | |
|---|---|---|---|---|---|---|---|---|
| OSF069128 | USA:CA | Raw Pistachios | PDS000027237 | 316 | Montevideo | SRR6109685 | 2015 | |
| OSF075603 | USA:CA | Pistachios | PDS000027237 | 316 | Montevideo | SRR6848181 | 2016 | |
| OSF075630 | USA:WA | Pistachios | PDS000027237 | 316 | Montevideo | SRR6782992 | 2016 | |
| OSF075637 | USA:CA | Pistachios | PDS000027237 | 316 | Montevideo | SRR6848879 | 2016 | |
| OSF075638 | USA:CA | Pistachios | PDS000027237 | 316 | Montevideo | SRR6848843 | 2016 | |
| OSF076741 | USA:CA | Pistachios | PDS000027237 | 316 | Montevideo | SRR6848188 | 2016 | |
| OSF076742 | USA:CA | Pistachios | PDS000027237 | 316 | Montevideo | SRR6848319 | 2016 | |
| OSF076748 | USA:CA | Pistachios | PDS000027237 | 316 | Montevideo | SRR6849216 | 2016 | |
| OSF076749 | USA:CA | Pistachios | PDS000027237 | 316 | Montevideo | SRR6849236 | 2016 | |
| OSF076750 | USA:CA | Pistachios | PDS000027237 | 316 | Montevideo | SRR6849235 | 2016 | G |
| OSF077989 | USA:CA | Environmental sponge | PDS000027237 | 316 | Montevideo | SRR6971655 | 2016 | |
| OSF077990 | USA:CA | Environmental sponge | PDS000027237 | 316 | Montevideo | SRR6967965 | 2016 | |
| OSF078698 | USA:CA | Environmental sponge | PDS000027237 | 316 | Montevideo | SRR7064464 | 2016 | |
| OSF084050 | USA:CA | Finished Almonds | PDS000027237 | 316 | Montevideo | SRR7638853 | 2017 | J |
| OSF088275 | USA:CA | Nuts | PDS000027237 | 316 | Montevideo | SRR8272032 | 2018 | G |
| OSF088276 | USA:CA | Nuts | PDS000027237 | 316 | Montevideo | SRR8272067 | 2018 | G |
| OSF088277 | USA:CA | Nuts | PDS000027237 | 316 | Montevideo | SRR8274725 | 2018 | G |
| OSF088278 | USA:CA | Nuts | PDS000027237 | 316 | Montevideo | SRR8274723 | 2018 | G |
| OSF088279 | USA:CA | Nuts | PDS000027237 | 316 | Montevideo | SRR8272153 | 2018 | G |
| OSF088282 | USA:CA | Nuts | PDS000027237 | 316 | Montevideo | SRR8272155 | 2018 | G |
| OSF088283 | USA:CA | Nuts | PDS000027237 | 316 | Montevideo | SRR8272614 | 2018 | G |
| PNUSAS001586 | USA | Clinical | PDS000027237 | 316 | Montevideo | SRR3277620 | Missing | |
| PNUSAS001648 | USA | Clinical | PDS000027237 | 316 | Montevideo | SRR3277288 | 2016 | |
| PNUSAS001685 | USA | Clinical | PDS000027237 | 316 | Montevideo | SRR3270925 | 2016 | |
| PNUSAS001723 | USA | Clinical | PDS000027237 | 316 | Montevideo | SRR3289850 | 2016 | |
| PNUSAS001724 | USA | Clinical | PDS000027237 | 316 | Montevideo | SRR3270998 | 2016 | |
| PNUSAS001745 | USA | Clinical | PDS000027237 | 316 | Montevideo | SRR3495148 | Missing | |
| PNUSAS001799 | USA | Clinical | PDS000027237 | 316 | Montevideo | SRR3289840 | 2016 | |
| PNUSAS001966 | USA | Clinical | PDS000027237 | 316 | Montevideo | SRR3473884 | 2016 | |
| PNUSAS002790 | USA | Clinical | PDS000027237 | 316 | Montevideo | SRR3897960 | 2016 | |
| PNUSAS004962 | USA | Clinical | PDS000027237 | 316 | Montevideo | SRR5031400 | 2016 | |
| PNUSAS008631 | USA | Clinical | PDS000027237 | 316 | Montevideo | SRR5336282 | Missing | |
| PNUSAS017345 | USA | Clinical | PDS000027237 | 316 | Montevideo | SRR5864604 | Missing | |
| PNUSAS034916 | USA | Clinical | PDS000027237 | 316 | Montevideo | SRR6870271 | 2018 | |
| PNUSAS041766 | USA | Clinical | PDS000027237 | 316 | Montevideo | SRR7235831 | Missing | |
| PNUSAS045784 | USA | Clinical | PDS000027237 | 316 | Montevideo | SRR7633156 | Missing | |
| PNUSAS049251 | USA | Clinical | PDS000027237 | 316 | Montevideo | SRR7688270 | Missing | |
| PNUSAS051029 | USA | Clinical | PDS000027237 | 316 | Montevideo | SRR7739852 | Missing | |
| PNUSAS136046 | USA | Clinical | PDS000027237 | 316 | Montevideo | SRR11174347 | Missing | |
| 12-1128 | Canada | Clinical | PDS000032600 | 138 | Montevideo | SRR5055291 | Missing | |
| CFSAN010209 | USA:CA | Pistachios | PDS000032600 | 138 | Montevideo | SRR5384655 | 2009 | A |
| FSIS31800836 | USA:UT | Beef | PDS000032600 | 138 | Montevideo | SRR7657490 | 2018 | |

| | | | | | | | | |
|---|---|---|---|---|---|---|---|---|
| FSLR9-1449 | USA:NY | Clinical | PDS000032600 | 138 | Montevideo | SRR8502795 | 2013 | |
| OSF005645 | USA:CA | Pistachios | PDS000032600 | 138 | Montevideo | SRR958039 | 2009 | G |
| OSF056829 | USA:CA | Raw Pistachios | PDS000032600 | 138 | Montevideo | SRR5120042 | 2014 | I |
| OSF067822 | USA:CA | Environmental sponge | PDS000032600 | 138 | Montevideo | SRR5991129 | 2017 | G |
| OSF069191 | USA:CA | Environmental sponge | PDS000032600 | 138 | Montevideo | SRR6109466 | 2015 | G |
| PNUSAS004495 | USA:WY | Cattle | PDS000032600 | 138 | Montevideo | SRR4418296 | Missing | |
| PNUSAS028544 | USA | Clinical | PDS000032600 | 138 | Montevideo | SRR6317156 | Missing | |
| PNUSAS044191 | USA | Clinical | PDS000032600 | 138 | Montevideo | SRR7429448 | Missing | |
| PNUSAS072017 | USA:MD | Clinical | PDS000032600 | 138 | Montevideo | SRR9972052 | Missing | |
| PNUSAS132560 | USA | Clinical | PDS000032600 | 138 | Montevideo | SRR11005270 | Missing | |
