# Supplementary material for: Genomic evidence of environmental and resident Salmonella Senftenberg and Montevideo contamination in the pistachio supply-chain
Source: PLoS One. 2021 Nov 4;16(11):e0259471. doi: 10.1371/journal.pone.0259471 (PMC8568146; doi:10.1371/journal.pone.0259471)
Supplement: S2 Table — SNP matrix generated from the CFSAN SNP pipeline for the Salmonella Senftenberg isolates from ST14. (PDF) [file pone.0259471.s002.pdf]

S2 Table: SNP matrix for ST14 Isolates

	CFSAN000622	SGSC2516	CFSAN015119	OSF067866	CFSAN016599	PNUSAS140610	CFSAN015122	OSF056593	CFSAN058295	OSF090575	OSF090574	FMA0102	OSF033320	PNUSAS001800	CFSAN045763	OSF059786	OSF059815	2016K-0180	OSF056809	OSF058109	CFSAN048428	OSF056360	OSF056362	OSF056361	OSF048660	CFSAN048429	CFSAN048427	OSF059119	OSF048786	OSF048599	OSF049332	CFSAN048426	OSF048658	OSF070289	OSF049331	OSF048659	PNUSAS008582	PNUSAS001664	OSF084195	OSF059253	OSF073577	OSF048657	OSF059289	2017K-0050	CFSAN047866	OSF047785	OSF033321	OSF059213	OSF073486	PNUSAS001739	OSF056838	OSF074224	OSF059265	CFSAN047567
CFSAN000622																																																						
SGSC2516	0																																																					
CFSAN015119	35	35																																																			
OSF067866	40	40	9																																																		
CFSAN016599	29	29	14	19																																																	
PNUSAS140610	29	29	14	19	8																																																
CFSAN015122	26	26	11	16	5	5																																															
OSF056593	26	26	11	16	5	5	2																																														
CFSAN058295	26	26	11	16	5	5	2	0																																													
OSF090575	28	28	13	18	7	7	4	2	2																																												
OSF090574	27	27	12	17	6	6	3	1	1	3																																											
FMA0102	27	27	12	17	6	6	3	3	3	5	4																																										
OSF033320	35	35	20	25	14	14	11	11	11	13	12	12																																									
PNUSAS001800	31	31	16	21	10	10	7	7	7	9	8	8	16																																								
CFSAN045763	27	27	13	18	7	7	4	4	4	6	5	5	13	9																																							
OSF059786	28	28	13	18	7	7	4	4	4	6	5	5	13	9	0																																						
OSF059815	29	29	15	20	9	9	6	6	6	8	7	7	15	11	2	2																																					
2016K-0180	36	36	21	26	15	15	12	12	12	14	13	13	21	17	8	8	10																																				
OSF056809	30	30	15	20	9	9	6	6	6	8	7	7	15	11	8	8	10	16																																			
OSF058109	32	32	17	22	11	11	8	8	8	10	9	9	17	13	10	10	12	18	10																																		
CFSAN048428	30	30	15	20	9	9	6	6	6	8	7	7	15	11	8	8	10	16	8	10																																		
OSF056360	35	35	20	25	14	14	11	11	11	13	12	12	20	16	13	13	15	21	13	15	5																																	
OSF056362	31	31	16	21	10	10	7	7	7	9	8	8	16	12	9	9	11	17	9	11	1	4																																
OSF056361	31	31	16	21	10	10	7	7	7	9	8	8	16	12	9	9	11	17	9	11	1	4	0																															
OSF048660	28	28	13	18	7	7	4	4	4	6	5	5	13	9	6	6	8	14	6	8	2	7	3	3																														
CFSAN048429	28	28	13	18	7	7	4	4	4	6	5	5	13	9	6	6	8	14	6	8	2	7	3	3	0																													
CFSAN048427	28	28	13	18	7	7	4	4	4	6	5	5	13	9	6	6	8	14	6	8	2	7	3	3	0	0																												
OSF059119	28	28	13	18	7	7	4	4	4	6	5	5	13	9	6	6	8	14	6	8	2	7	3	3	0	0	0																											
OSF048786	27	27	13	18	7	7	4	4	4	6	5	5	13	9	6	6	8	14	6	8	2	7	3	3	0	0	0	0																										
OSF048599	37	37	22	27	16	16	13	13	13	15	14	14	22	18	15	15	17	23	15	17	11	16	12	12	9	9	9	9	8																									
OSF049332	29	29	15	20	9	9	6	6	6	8	7	7	15	11	8	8	10	16	8	10	4	9	5	5	2	2	2	2	2	7																								
CFSAN048426	30	30	15	20	9	9	6	6	6	8	7	7	15	11	8	8	10	16	8	10	4	9	5	5	2	2	2	2	2	7	0																							
OSF048658	30	30	15	20	9	9	6	6	6	8	7	7	15	11	8	8	10	16	8	10	4	9	5	5	2	2	2	2	2	7	0	0																						
OSF070289	30	30	15	20	9	9	6	6	6	8	7	7	15	11	8	8	10	16	8	10	4	9	5	5	2	2	2	2	2	7	0	0	0																					
OSF049331	30	30	15	20	9	9	6	6	6	8	7	7	15	11	8	8	10	16	8	10	4	9	5	5	2	2	2	2	2	7	0	0	0	0																				
OSF048659	30	30	15	20	9	9	6	6	6	8	7	7	15	11	8	8	10	16	8	10	4	9	5	5	2	2	2	2	2	7	0	0	0	0	0																			
PNUSAS008582	29	29	14	19	8	8	5	5	5	7	6	6	14	10	7	7	9	15	7	9	3	8	4	4	1	1	1	1	1	10	3	3	3	3	3	3																		
PNUSAS001664	29	29	14	19	8	8	5	5	5	7	6	6	14	10	7	7	9	15	7	9	3	8	4	4	1	1	1	1	1	10	3	3	3	3	3	3	2																	
OSF084195	29	29	14	19	8	8	5	5	5	7	6	6	14	10	7	7	9	15	7	9	3	8	4	4	1	1	1	1	1	10	3	3	3	3	3	3	2	2																
OSF059253	29	29	14	19	8	8	5	5	5	7	6	6	14	10	7	7	9	15	7	9	3	8	4	4	1	1	1	1	1	10	3	3	3	3	3	3	2	2	2															
OSF073577	29	29	14	19	8	8	5	5	5	7	6	6	14	9	7	7	9	15	7	9	3	8	4	4	1	1	1	1	1	10	3	3	3	3	3	3	2	2	2	0														
OSF048657	30	30	15	20	9	9	6	6	6	8	7	7	15	11	8	8	10	16	8	10	4	9	5	5	2	2	2	2	2	11	4	4	4	4	4	4	3	3	3	3	3													
OSF059289	31	31	16	21	10	10	7	7	7	9	8	8	16	12	9	9	11	17	9	11	5	10	6	6	3	3	3	3	3	12	5	5	5	5	5	5	4	4	4	4	4	1												
2017K-0050	40	40	26	31	20	20	17	17	17	19	18	18	26	22	19	19	21	27	19	21	15	20	16	16	13	13	13	13	13	22	15	15	15	15	15	15	14	14	14	14	13	15	16											
CFSAN047866	31	31	16	21	10	10	7	7	7	9	8	8	16	12	9	9	11	17	9	11	5	10	6	6	3	3	3	3	3	12	5	5	5	5	5	5	4	4	4	4	4	5	6	14										
OSF047785	31	31	16	21	10	10	7	7	7	9	8	8	16	12	9	9	11	17	9	11	5	10	6	6	3	3	3	3	3	12	5	5	5	5	5	5	4	4	4	4	4	5	6	14	0									
OSF033321	29	29	14	19	8	8	5	5	5	7	6	6	14	10	7	7	9	15	7	9	3	8	4	4	1	1	1	1	1	10	3	3	3	3	3	3	2	2	2	2	2	3	4	14	4	4								
OSF059213	33	33	18	23	12	12	9	9	9	11	10	10	18	14	11	11	13	19	11	13	7	12	8	8	5	5	5	5	5	14	7	7	7	7	7	7	6	6	6	6	6	7	8	18	8	8	4							
OSF073486	33	33	18	23	12	12	9	9	9	11	10	10	18	14	11	11	13	19	11	13	7	12	8	8	5	5	5	5	5	14	7	7	7	7	7	7	6	6	6	6	6	7	8	18	8	8	4	0						
PNUSAS001739	31	31	16	21	10	10	7	7	7	9	8	8	16	12	9	9	11	17	9	11	5	10	6	6	3	3	3	3	3	12	5	5	5	5	5	5	4	4	4	4	4	5	6	16	6	6	2	6	6					
OSF056838	32	32	17	22	11	11	8	8	8	10	9	9	17	13	10	10	12	18	10	12	6	11	7	7	4	4	4	4	4	13	6	6	6	6	6	6	5	5	5	5	5	6	7	17	7	7	3	7	7	3				
OSF074224	30	30	15	20	9	9	6	6	6	8	7	7	15	11	8	8	10	16	8	10	4	9	5	5	2	2	2	2	2	11	4	4	4	4	4	4	3	3	3	3	3	4	5	15	5	5	1	5	5	1	2			
OSF059265	30	30	15	20	9	9	6	6	6	8	7	7	15	11	8	8	10	16	8	10	4	9	5	5	2	2	2	2	2	11	4	4	4	4	4	4	3	3	3	3	3	4	5	15	5	5	1	5	5	1	2	0		
CFSAN047567	30	30	15	20	9	9	6	6	6	8	7	7	15	11	8	8	10	16	8	10	4	9	5	5	2	2	2	2	2	11	4	4	4	4	4	4	3	3	3	3	3	4	5	15	5	5	1	5	5	1	2	0	0	
