# Supplementary material for: Genomic evidence of environmental and resident Salmonella Senftenberg and Montevideo contamination in the pistachio supply-chain
Source: PLoS One. 2021 Nov 4;16(11):e0259471. doi: 10.1371/journal.pone.0259471 (PMC8568146; doi:10.1371/journal.pone.0259471)
Supplement: S3 Table — SNP matrix generated from the CFSAN SNP pipeline for the Salmonella Senftenberg isolates from ST185. (PDF) [file pone.0259471.s003.pdf]

S3 Table: SNP matrix for ST185 Isolates

	FSE0085	FSW0104	FSW0103	2013K-0731	2013K-0735	2013K-0777	FSF102	FNW19i50	FNW19i51	CFSAN032229	CFSAN032231	CFSAN032230	CFSAN017737	CFSAN017738	CFSAN017739	CFSAN017740	CFSAN017741	CFSAN017744	CFSAN017745	CFSAN017746	CFSAN017747	CFSAN017748	CFSAN017749	CFSAN017750	CFSAN017751	CFSAN017752	CFSAN017753	CFSAN017754	OSF056675	OSF056676	OSF056674	OSF056673	OSF056753	OSF056767	OSF056768	OSF056769	OSF057938	OSF057945	OSF057940	OSF057941	OSF057942	OSF057943	OSF057939	OSF057981	OSF057947	OSF057951	OSF057979	OSF057948	OSF057980	OSF057946	OSF057982	OSF057978	CFSAN086744	OSF087751	CFSAN087304
FSE0085																																																							
FSW0104	75																																																						
FSW0103	76	1																																																					
2013K-0731	75	1	2																																																				
2013K-0735	74	0	1	1																																																			
2013K-0777	75	0	1	1	0																																																		
FSF102	76	1	2	2	1	1																																																	
FNW19i50	75	0	1	1	0	0	1																																																
FNW19i51	75	0	1	1	0	0	1	0																																															
CFSAN032229	76	1	2	2	1	1	2	1	1																																														
CFSAN032231	78	4	5	5	4	4	5	4	4	5																																												
CFSAN032230	75	0	1	1	0	0	1	0	0	1	4																																											
CFSAN017737	75	0	1	1	0	0	1	0	0	1	4	0																																										
CFSAN017738	76	1	2	2	1	1	2	1	1	2	5	1	1																																									
CFSAN017739	75	0	1	1	0	0	1	0	0	1	4	0	0	1																																								
CFSAN017740	75	0	1	1	0	0	1	0	0	1	4	0	0	1	0																																							
CFSAN017741	77	2	3	3	2	2	3	2	2	3	6	2	2	3	2	2																																						
CFSAN017744	75	0	1	1	0	0	1	0	0	1	4	0	0	1	0	0	2																																					
CFSAN017745	75	0	1	1	0	0	1	0	0	1	4	0	0	1	0	0	2	0																																				
CFSAN017746	75	0	1	1	0	0	1	0	0	1	4	0	0	1	0	0	2	0	0																																			
CFSAN017747	75	0	1	1	0	0	1	0	0	1	4	0	0	1	0	0	2	0	0	0																																		
CFSAN017748	77	1	3	3	2	2	3	2	2	3	5	2	2	3	2	2	3	2	2	1	2																																	
CFSAN017749	75	0	1	1	0	0	1	0	0	1	4	0	0	1	0	0	2	0	0	0	0	2																																
CFSAN017750	75	0	1	1	0	0	1	0	0	1	4	0	0	1	0	0	2	0	0	0	0	1	0																															
CFSAN017751	76	1	2	2	1	1	2	1	1	2	5	1	1	2	1	1	3	1	1	1	1	2	1	1																														
CFSAN017752	75	0	1	1	0	0	1	0	0	1	4	0	0	1	0	0	2	0	0	0	0	2	0	0	1																													
CFSAN017753	75	0	1	1	0	0	1	0	0	1	4	0	0	1	0	0	2	0	0	0	0	1	0	0	1	0																												
CFSAN017754	76	1	2	2	1	1	0	1	1	2	5	1	1	2	1	1	3	1	1	1	1	3	1	1	2	1	1																											
OSF056675	75	0	1	1	0	0	1	0	0	1	4	0	0	1	0	0	2	0	0	0	0	2	0	0	1	0	0	1																										
OSF056676	77	2	3	3	2	2	1	2	2	3	6	2	2	3	2	2	4	2	2	2	2	4	2	2	3	2	2	1	2																									
OSF056674	76	1	2	2	1	1	0	1	1	2	5	1	1	2	1	1	3	1	1	1	1	3	1	1	2	1	1	0	1	1																								
OSF056673	75	0	1	1	0	0	1	0	0	1	4	0	0	1	0	0	2	0	0	0	0	2	0	0	1	0	0	1	0	2	1																							
OSF056753	75	0	1	1	0	0	1	0	0	1	4	0	0	1	0	0	2	0	0	0	0	2	0	0	1	0	0	1	0	2	1	0																						
OSF056767	75	0	1	1	0	0	1	0	0	1	4	0	0	1	0	0	2	0	0	0	0	2	0	0	1	0	0	1	0	2	1	0	0																					
OSF056768	76	2	3	3	2	2	3	2	2	3	6	2	2	3	2	2	4	2	2	2	2	4	2	2	3	2	2	3	2	4	3	2	2	2																				
OSF056769	75	0	1	1	0	0	1	0	0	1	4	0	0	1	0	0	2	0	0	0	0	2	0	0	1	0	0	1	0	2	1	0	0	0	2																			
OSF057938	77	2	3	3	2	2	3	2	2	3	6	2	2	3	2	2	4	2	2	2	2	4	2	2	3	2	2	3	2	4	3	2	2	2	4	2																		
OSF057945	77	2	3	3	2	2	3	2	2	3	6	2	2	3	2	2	4	2	2	2	2	4	2	2	3	2	2	3	2	4	3	2	2	2	4	2	2																	
OSF057940	71	0	1	1	0	0	1	0	0	1	4	0	0	1	0	0	2	0	0	0	0	2	0	0	1	0	0	1	0	2	1	0	0	0	2	0	2	2																
OSF057941	76	4	5	5	4	4	5	4	4	5	8	4	4	5	4	4	6	4	4	4	4	6	4	4	5	4	4	5	4	6	5	4	4	4	6	4	6	6	4															
OSF057942	75	0	1	1	0	0	1	0	0	1	4	0	0	1	0	0	2	0	0	0	0	2	0	0	1	0	0	1	0	2	1	0	0	0	2	0	2	2	0	4														
OSF057943	76	1	2	2	1	1	2	1	1	2	5	1	1	2	1	1	3	1	1	1	1	3	1	1	2	1	1	2	1	3	2	1	1	1	3	1	3	3	1	5	1													
OSF057939	74	0	1	1	0	0	1	0	0	1	4	0	0	1	0	0	2	0	0	0	0	2	0	0	1	0	0	1	0	2	1	0	0	0	2	0	2	2	0	4	0	1												
OSF057981	76	1	2	2	1	1	0	1	1	2	5	1	1	2	1	1	3	1	1	1	1	3	1	1	2	1	1	0	1	1	0	1	1	1	3	1	3	3	1	5	1	2	1											
OSF057947	76	1	2	2	1	1	2	1	1	2	5	1	1	2	1	1	3	1	1	1	1	3	1	1	2	1	1	2	1	3	2	1	1	1	3	1	1	1	1	5	1	2	1	2										
OSF057951	74	0	1	1	0	0	1	0	0	1	4	0	0	1	0	0	2	0	0	0	0	2	0	0	1	0	0	1	0	2	1	0	0	0	2	0	2	2	0	4	0	1	0	1	1									
OSF057979	75	0	1	1	0	0	1	0	0	1	4	0	0	1	0	0	2	0	0	0	0	2	0	0	1	0	0	1	0	2	1	0	0	0	2	0	2	2	0	4	0	1	0	1	1	0								
OSF057948	76	1	2	2	1	1	2	1	1	2	5	1	1	2	1	1	3	1	1	1	1	3	1	1	2	1	1	2	1	3	2	1	1	1	3	1	3	3	1	5	1	2	1	2	2	1	1							
OSF057980	75	0	1	1	0	0	1	0	0	1	4	0	0	1	0	0	2	0	0	0	0	2	0	0	1	0	0	1	0	2	1	0	0	0	2	0	2	2	0	4	0	1	0	1	1	0	0	1						
OSF057946	76	1	2	2	1	1	2	1	1	2	5	1	1	2	1	1	3	1	1	1	1	3	1	1	2	1	1	2	1	3	2	1	1	1	3	1	3	3	1	5	1	2	1	2	2	1	1	2	1					
OSF057982	76	1	2	2	1	1	2	1	1	2	5	1	1	2	1	1	3	1	1	1	1	3	1	1	2	1	1	2	1	3	2	1	1	1	3	1	3	3	1	5	1	2	1	2	2	1	1	2	1	2				
OSF057978	76	1	2	2	1	1	2	1	1	2	5	1	1	2	1	1	3	1	1	1	1	3	1	1	2	1	1	2	1	3	2	1	1	1	3	1	3	3	1	5	1	2	1	2	2	1	1	2	1	2	2			
CFSAN086744	78	3	4	4	3	3	4	3	3	4	7	3	3	4	3	3	5	3	3	3	3	5	3	3	4	3	3	4	3	5	4	3	3	3	5	3	5	5	3	7	3	4	3	4	4	3	3	4	3	4	4	4		
OSF087751	79	4	5	5	4	4	5	4	4	5	8	4	4	5	4	4	6	4	4	4	4	6	4	4	5	4	4	5	4	6	5	4	4	4	6	4	6	6	4	8	4	5	4	5	5	4	4	5	4	5	5	5	3	
CFSAN087304	78	3	4	4	3	3	4	3	3	4	7	3	3	4	3	3	5	3	3	3	3	4	3	3	4	3	3	4	3	5	4	3	3	3	5	3	5	5	3	7	3	4	3	4	4	3	3	4	3	4	4	4	2	1	
