# Supplementary material for: Genomic evidence of environmental and resident Salmonella Senftenberg and Montevideo contamination in the pistachio supply-chain
Source: PLoS One. 2021 Nov 4;16(11):e0259471. doi: 10.1371/journal.pone.0259471 (PMC8568146; doi:10.1371/journal.pone.0259471)
Supplement: S4 Table — SNP matrix generated from the CFSAN SNP pipeline for the Salmonella Montevideo isolates from ST316. (PDF) [file pone.0259471.s004.pdf]

S4 Table: SNP matrix for ST316 Isolates

	FSIS1709808	FSIS1606641	FSL_R8-4922	OSF077989	OSF084050	CFSAN064799	CFSAN016380	CFSAN015113	CFSAN015123	CFSAN016412	CFSAN015120	CFSAN013604	CFSAN016600	315731156	CFSAN014882	CFSAN016601	CFSAN015114	PNUSAS001966	44713	FSL_R8-4916	OSF045712	CFSAN015118	CFSAN016379	PNUSAS049251	CFSAN015115	2016K-0167	PNUSAS001799	CFSAN037640	OSF051202	708223	OSF059120	PNUSAS001745	CFSAN015117	OSF046987	CFSAN015116	CFSAN047168	CFSAN047169	CFSAN051296	OSF047721	CFSAN016411	PNUSAS001586	CFSAN000244	OSF056837	PNUSAS034916	OSF069128	PNUSAS136046	OSF078698	378603	PNUSAS051029	PNUSAS001648	533421	570824	PNUSAS045784	PNUSAS041766	PNUSAS008631	PNUSAS017345	PNUSAS001724	PNUSAS001685	CFSAN045764	PNUSAS002790	PNUSAS004962	PNUSAS001723	OSF088277	OSF075638	OSF076748	OSF075630	OSF075603	CFSAN047166	CFSAN047167	OSF075637	OSF069055	OSF076742	OSF088279	OSF076750	OSF077990	OSF076749	OSF088276	OSF088275	OSF076741	OSF088278	OSF088283	OSF088282
FSIS1709808
FSIS1606641	9
FSL_R8-4922	27	28
OSF077989	25	26	4
OSF084050	24	25	3	1
CFSAN064799	24	25	3	1	0
CFSAN016380	24	25	3	1	0	0
CFSAN015113	24	25	3	1	0	0	0
CFSAN015123	23	24	4	2	1	1	1	1
CFSAN016412	23	24	4	2	1	1	1	1	0
CFSAN015120	23	24	4	2	1	1	1	1	0	0
CFSAN013604	23	24	4	2	1	1	1	1	0	0	0
CFSAN016600	23	24	4	2	1	1	1	1	0	0	0	0
315731156	23	24	4	2	1	1	1	1	0	0	0	0	0
CFSAN014882	23	24	4	2	1	1	1	1	0	0	0	0	0	0
CFSAN016601	25	26	6	4	3	3	3	3	2	2	2	2	2	2	2
CFSAN015114	25	26	6	4	3	3	3	3	2	2	2	2	2	2	2	4
PNUSAS001966	26	27	7	5	4	4	4	4	3	3	3	3	3	3	3	5	5
44713	24	25	5	3	2	2	2	2	1	1	1	1	1	1	1	3	3	2
FSL_R8-4916	26	27	7	5	4	4	4	4	3	3	3	3	3	3	3	5	5	6	4
OSF045712	26	27	7	5	4	4	4	4	2	3	3	3	3	3	3	5	5	6	4	6
CFSAN015118	24	25	5	3	1	2	2	2	1	1	1	1	1	0	0	3	3	4	1	4	4
CFSAN016379	24	25	5	3	2	2	2	2	1	1	1	1	1	1	1	3	3	4	2	4	4	2
PNUSAS049251	27	28	8	6	5	5	5	5	4	4	4	4	4	4	4	6	6	7	5	7	7	5	5
CFSAN015115	24	25	5	3	2	2	2	2	1	1	1	1	1	1	1	3	3	4	2	4	4	2	2	3
2016K-0167	24	25	5	3	2	2	2	2	1	1	1	1	1	1	1	3	3	4	2	4	4	2	2	3	0
PNUSAS001799	24	25	5	3	2	2	2	2	1	1	1	1	1	1	1	3	3	4	2	4	4	2	2	3	0	0
CFSAN037640	24	25	5	3	2	2	2	2	1	1	1	1	1	1	1	3	3	4	2	4	4	2	2	3	0	0	0
OSF051202	24	25	5	3	2	2	2	2	1	1	1	1	1	1	1	3	3	4	2	4	4	2	2	3	0	0	0	0
708223	24	25	5	3	2	2	2	2	1	1	1	1	1	1	1	3	3	4	2	4	4	2	2	3	0	0	0	0	0
OSF059120	24	25	5	3	2	2	2	2	1	1	1	1	1	1	1	3	3	4	2	4	4	2	2	3	0	0	0	0	0	0
PNUSAS001745	24	25	5	3	2	2	2	2	1	1	1	1	1	1	1	3	3	4	2	4	4	2	2	3	0	0	0	0	0	0	0
CFSAN015117	24	25	5	3	2	2	2	2	1	1	1	1	1	1	1	3	3	4	2	4	4	2	2	3	0	0	0	0	0	0	0	0
OSF046987	24	25	5	3	2	2	2	2	1	1	1	1	1	1	1	3	3	4	2	4	4	2	2	3	0	0	0	0	0	0	0	0	0
CFSAN015116	24	25	5	3	2	2	2	2	1	1	1	1	1	1	1	3	3	4	2	4	4	2	2	3	0	0	0	0	0	0	0	0	0	0
CFSAN047168	24	25	5	3	2	2	2	2	1	1	1	1	1	1	1	3	3	4	2	4	4	2	2	3	0	0	0	0	0	0	0	0	0	0	0
CFSAN047169	24	25	5	3	2	2	2	2	1	1	1	1	1	1	1	3	3	4	2	4	4	2	2	3	0	0	0	0	0	0	0	0	0	0	0	0
CFSAN051296	24	25	5	3	2	2	2	2	1	1	1	1	1	1	1	3	3	4	2	4	4	1	2	3	0	0	0	0	0	0	0	0	0	0	0	0	0
OSF047721	24	25	5	2	2	2	2	2	1	1	1	1	1	1	1	3	3	4	2	4	4	1	2	3	0	0	0	0	0	0	0	0	0	0	0	0	0	0
CFSAN016411	25	26	6	4	3	3	3	3	2	2	2	2	2	2	2	4	4	5	3	5	5	3	3	4	1	1	1	1	1	1	1	1	1	1	1	1	1	1	1
PNUSAS001586	25	26	6	4	3	3	3	3	2	2	2	2	2	2	2	4	4	5	3	5	5	3	3	4	1	1	1	1	1	1	1	1	1	1	1	1	1	1	1	2
CFSAN000244	25	26	6	4	3	3	3	3	2	2	2	2	2	2	2	4	4	5	3	5	5	2	3	4	1	1	1	1	1	1	1	1	1	1	1	1	1	1	1	2	2
OSF056837	25	26	6	4	3	3	3	3	2	2	2	2	2	2	2	4	4	5	3	5	5	2	3	4	1	1	1	1	1	1	1	1	1	1	1	1	1	1	1	2	2	2
PNUSAS034916	26	27	7	5	4	4	4	4	3	3	3	3	3	3	3	5	5	6	4	6	6	4	4	5	2	2	2	2	2	2	2	2	2	2	2	2	2	2	2	3	3	3	3
OSF069128	26	27	7	5	4	4	4	4	3	3	3	3	3	3	3	5	5	6	4	6	6	4	4	5	2	2	2	2	2	2	2	2	2	2	2	2	2	2	2	3	3	3	3	4
PNUSAS136046	26	27	7	5	4	4	4	4	3	3	3	3	3	3	3	5	5	6	4	6	6	4	4	5	2	2	2	2	2	2	2	2	2	2	2	2	2	2	2	3	3	3	3	4	4
OSF078698	26	27	7	5	4	4	4	4	3	3	3	3	3	3	3	5	5	6	4	6	6	4	4	5	2	2	2	2	2	2	2	2	2	2	2	2	2	2	2	3	3	3	3	4	4	4
378603	27	28	8	6	5	5	5	5	4	4	4	4	4	4	4	6	6	7	5	7	7	4	5	6	3	3	3	3	3	3	3	3	3	3	3	3	3	3	3	4	4	4	4	5	5	5	5
PNUSAS051029	27	28	8	5	5	5	5	5	4	4	4	4	4	4	4	6	6	7	5	7	7	5	5	6	3	3	3	3	3	3	3	3	3	3	3	3	3	3	3	4	4	4	4	5	5	5	5	6
PNUSAS001648	27	28	8	6	5	5	5	5	4	4	4	4	4	4	4	6	6	7	5	7	7	5	5	6	3	3	3	3	3	3	3	3	3	3	3	3	3	3	3	4	4	4	4	5	5	5	5	6	6
533421	27	28	8	6	5	5	5	5	4	4	4	4	4	4	4	6	6	7	5	7	7	5	5	6	3	3	3	3	3	3	3	3	3	3	3	3	3	3	3	4	4	4	4	5	5	5	5	6	6	6
570824	28	29	9	7	6	6	6	6	5	5	5	5	5	5	5	7	7	8	6	8	8	6	6	7	4	4	4	4	4	4	4	4	4	4	4	4	4	4	4	5	5	5	5	6	6	6	6	7	7	7	1
PNUSAS045784	26	27	7	5	4	4	4	4	3	3	3	3	3	3	3	5	5	6	4	6	6	4	4	5	2	2	2	2	2	2	2	2	2	2	2	2	2	2	2	3	3	3	3	4	4	4	4	5	5	5	5	6
PNUSAS041766	26	27	7	5	4	4	4	4	3	3	3	3	3	3	3	5	5	6	4	6	6	3	4	5	2	2	2	2	2	2	2	2	2	2	2	2	2	2	2	3	3	3	3	4	4	4	4	5	5	5	5	6	0
PNUSAS008631	26	27	7	5	4	4	4	4	3	3	3	3	3	3	3	5	5	6	4	6	6	4	4	5	2	2	2	2	2	2	2	2	2	2	2	2	2	2	2	3	3	3	3	4	4	4	4	5	5	5	5	6	4	4
PNUSAS017345	34	35	15	13	12	12	12	12	11	11	11	11	11	11	11	13	13	14	12	14	14	11	12	13	10	10	10	10	10	10	10	10	10	10	10	10	10	10	10	11	11	11	11	12	11	12	12	13	13	13	13	14	12	12	8
PNUSAS001724	26	27	7	5	4	4	4	4	3	3	3	3	3	3	3	5	5	6	4	6	6	4	4	5	2	2	2	2	2	2	2	2	2	2	2	2	2	2	2	3	3	3	3	4	4	4	4	5	5	5	5	6	4	4	4	12
PNUSAS001685	26	27	7	5	4	4	4	4	3	3	3	3	3	3	3	5	5	6	4	6	6	4	4	5	2	2	2	2	2	2	2	2	2	2	2	2	2	2	2	3	3	3	3	4	4	4	4	5	5	5	5	6	4	4	4	12	2
CFSAN045764	25	26	6	4	3	3	3	3	2	2	2	2	2	2	2	4	4	5	3	5	5	2	3	4	1	1	1	1	1	1	1	1	1	1	1	1	1	1	1	2	2	2	2	3	3	3	3	4	4	4	4	5	3	3	3	11	1	1
PNUSAS002790	25	26	6	4	3	3	3	3	2	2	2	2	2	2	2	4	4	5	3	5	5	3	3	4	1	1	1	1	1	1	1	1	1	1	1	1	1	1	1	2	2	2	2	3	3	3	3	4	4	4	4	5	3	3	3	11	3	3	2
PNUSAS004962	25	26	6	4	3	3	3	3	2	2	2	2	2	2	2	4	4	5	3	5	5	3	3	4	1	1	1	1	1	1	1	1	1	1	1	1	1	1	1	2	2	2	2	3	3	3	3	4	4	4	4	5	3	3	3	11	3	3	2	0
PNUSAS001723	30	31	11	9	8	8	8	8	7	7	7	7	7	7	7	9	9	10	8	10	10	8	8	9	6	6	6	6	6	6	6	6	6	6	6	6	6	6	6	7	7	7	7	8	8	8	8	9	9	9	9	10	8	8	8	16	8	8	7	7	7
OSF088277	27	28	8	6	5	5	5	5	4	4	4	4	4	4	4	6	6	7	5	7	7	5	5	6	3	3	3	3	3	3	3	3	3	3	3	3	3	3	3	4	4	4	4	5	5	5	5	6	6	6	6	7	5	5	5	13	5	5	4	4	4	7
OSF075638	27	28	8	6	5	5	5	5	4	4	4	4	4	4	4	6	6	7	5	7	7	5	5	6	3	3	3	3	3	3	3	3	3	3	3	3	3	3	3	4	4	4	4	5	5	5	5	6	6	6	6	7	5	5	5	13	5	5	4	4	4	7	4
OSF076748	30	31	11	9	8	8	8	8	6	7	7	7	7	7	7	9	9	10	8	10	10	8	8	9	6	6	6	6	6	6	6	6	6	6	6	6	6	6	6	7	7	7	7	8	8	8	8	9	9	9	9	10	8	8	8	16	8	8	7	7	7	10	7	5
OSF075630	30	31	11	9	8	8	8	8	6	7	7	7	7	7	7	9	9	10	8	10	10	8	8	9	6	6	6	6	6	6	6	6	6	6	6	6	6	6	6	7	7	7	7	8	8	8	8	9	9	9	9	10	8	8	8	16	8	8	7	7	7	10	7	5	0
OSF075603	30	31	11	9	8	8	8	8	6	7	7	7	7	7	7	9	9	10	8	10	10	8	8	9	6	6	6	6	6	6	6	6	6	6	6	6	6	6	6	7	7	7	7	8	8	8	8	9	9	9	9	10	8	8	8	16	8	8	7	7	7	10	7	5	0	0
CFSAN047166	26	27	7	5	4	4	4	4	3	3	3	3	3	3	3	5	5	6	4	6	6	4	4	5	2	2	2	2	2	2	2	2	2	2	2	2	2	2	2	3	3	3	3	4	4	4	4	5	5	5	5	6	4	4	4	12	4	4	3	3	3	6	3	1	4	4	4
CFSAN047167	26	27	7	5	4	4	4	4	3	3	3	3	3	3	3	5	5	6	4	6	6	4	4	5	2	2	2	2	2	2	2	2	2	2	2	2	2	2	2	3	3	3	3	4	4	4	4	5	5	5	5	6	4	4	4	12	4	4	3	3	3	6	3	1	4	4	4	0
OSF075637	26	27	7	5	4	4	4	4	3	3	3	3	3	3	3	5	5	6	4	6	6	4	4	5	2	2	2	2	2	2	2	2	2	2	2	2	2	2	2	3	3	3	3	4	4	4	4	5	5	5	5	6	4	4	4	12	4	4	3	3	3	6	3	1	4	4	4	0	0
OSF069055	26	27	7	5	4	4	4	4	3	3	3	3	3	3	3	5	5	6	4	6	6	4	4	5	2	2	2	2	2	2	2	2	2	2	2	2	2	2	2	3	3	3	3	4	4	4	4	5	5	5	5	6	4	4	4	12	4	4	3	3	3	6	3	1	4	4	4	0	0	0
OSF076742	25	26	6	4	3	3	3	3	2	2	2	2	2	2	2	4	4	5	3	5	5	3	3	4	1	1	1	1	1	1	1	1	1	1	1	1	1	1	1	2	2	2	2	3	3	3	3	4	4	4	4	5	3	3	3	11	3	3	2	2	2	5	2	2	5	5	5	1	1	1	1
OSF088279	25	26	6	4	3	3	3	3	2	2	2	2	2	2	2	4	4	5	3	5	5	3	3	4	1	1	1	1	1	1	1	1	1	1	1	1	1	1	1	2	2	2	2	3	3	3	3	4	4	4	4	5	3	3	3	11	3	3	2	2	2	5	2	2	5	5	5	1	1	1	1	0
OSF076750	25	26	6	4	3	3	3	3	2	2	2	2	2	2	2	4	4	5	3	5	5	2	3	4	1	1	1	1	1	1	1	1	1	1	1	1	1	1	1	2	2	2	2	3	3	3	3	4	4	4	4	5	3	3	3	11	3	3	2	2	2	5	2	2	5	5	5	1	1	1	1	0	0
OSF077990	25	26	6	4	3	3	3	3	2	2	2	2	2	2	2	4	4	5	3	5	5	2	3	4	1	1	1	1	1	1	1	1	1	1	1	1	1	1	1	2	2	2	2	3	3	3	3	4	4	4	4	5	3	3	3	11	3	3	2	2	2	5	2	2	5	5	5	1	1	1	1	0	0	0
OSF076749	25	26	6	4	3	3	3	3	2	2	2	2	2	2	2	4	4	5	3	5	5	2	3	4	1	1	1	1	1	1	1	1	1	1	1	1	1	1	1	2	2	2	2	3	3	3	3	4	4	4	4	5	3	3	3	11	3	3	2	2	2	5	2	2	5	5	5	1	1	1	1	0	0	0	0
OSF088276	26	27	7	5	4	4	4	4	3	3	3	3	3	3	3	5	5	6	4	6	6	3	4	5	2	2	2	2	2	2	2	2	2	2	2	2	2	2	2	3	3	3	3	4	4	4	4	5	5	5	5	6	4	4	4	12	4	4	3	3	3	6	3	3	6	6	6	2	2	2	2	1	1	1	1	1
OSF088275	26	27	7	5	4	4	4	4	3	3	3	3	3	3	3	5	5	6	4	6	6	3	4	5	2	2	2	2	2	2	2	2	2	2	2	2	2	2	2	3	3	3	3	4	4	4	4	5	5	5	5	6	4	4	4	12	4	4	3	3	3	6	3	3	6	6	6	2	2	2	2	1	1	1	1	1	2
OSF076741	26	27	7	5	4	4	4	4	3	3	3	3	3	3	3	5	5	6	4	6	6	3	4	5	2	2	2	2	2	2	2	2	2	2	2	2	2	2	2	3	3	3	3	4	4	4	4	5	5	5	5	6	4	4	4	12	4	4	3	3	3	6	3	3	6	6	6	2	2	2	2	1	1	1	1	1	2	2
OSF088278	26	27	7	5	4	4	4	4	3	3	3	3	3	3	3	5	5	6	4	6	6	3	4	5	2	2	2	2	2	2	2	2	2	2	2	2	2	2	2	3	3	3	3	4	4	4	4	5	5	5	5	6	4	4	4	12	4	4	3	3	3	6	3	3	6	6	6	2	2	2	2	1	1	1	1	1	2	2	2
OSF088283	26	27	7	5	4	4	4	4	3	3	3	3	3	3	3	5	5	6	4	6	6	3	4	5	2	2	2	2	2	2	2	2	2	2	2	2	2	2	2	3	3	3	3	4	4	4	4	5	5	5	5	6	4	4	4	12	4	4	3	3	3	6	3	3	6	6	6	2	2	2	2	1	1	1	1	1	2	2	2	2
OSF088282	26	27	7	5	4	4	4	4	3	3	3	3	3	3	3	5	5	6	4	6	6	3	4	5	2	2	2	2	2	2	2	2	2	2	2	2	2	2	2	3	3	3	3	4	4	4	4	5	5	5	5	6	4	4	4	12	4	4	3	3	3	6	3	3	6	6	6	2	2	2	2	1	1	1	1	1	2	2	2	2	2
