# Supplementary material for: Genomic evidence of environmental and resident Salmonella Senftenberg and Montevideo contamination in the pistachio supply-chain
Source: PLoS One. 2021 Nov 4;16(11):e0259471. doi: 10.1371/journal.pone.0259471 (PMC8568146; doi:10.1371/journal.pone.0259471)
Supplement: S5 Table — SNP matrix generated from the CFSAN SNP pipeline for the Salmonella Montevideo isolates from ST138. (PDF) [file pone.0259471.s005.pdf]

S5 Table: SNP matrix for ST138 Isolates

	PNUSAS004495	PNUSAS044191	FSIS31800836	PNUSAS072017	PNUSAS028544	PNUSAS132560	CFSAN010209	FSLR9-1449	OSF056829	OSF067822	OSF005645	12-1128	OSF069191
PNUSAS004495													
PNUSAS044191	18												
FSIS31800836	31	22											
PNUSAS072017	38	32	44										
PNUSAS028544	42	35	47	7									
PNUSAS132560	36	30	43	30	34								
CFSAN010209	37	31	44	31	35	7							
FSLR9-1449	38	32	44	32	35	8	3						
OSF056829	38	32	45	32	36	8	3	2					
OSF067822	37	31	44	32	36	8	3	2	2				
OSF005645	36	30	42	30	33	6	1	2	2	2			
12-1128	36	30	43	30	34	7	2	3	3	3	1		
OSF069191	38	32	45	32	36	8	3	4	4	4	2	3	
