# Supplementary material for: Genomic evidence of environmental and resident Salmonella Senftenberg and Montevideo contamination in the pistachio supply-chain
Source: PLoS One. 2021 Nov 4;16(11):e0259471. doi: 10.1371/journal.pone.0259471 (PMC8568146; doi:10.1371/journal.pone.0259471)
Supplement: S6 Table — This table shows the additional phages that were identified by PHASTER in the closed genomes of Salmonella Senftenberg and Montevideo from our study. (PDF) [file pone.0259471.s006.pdf]

S6 Table: Phages identified in study isolates:

Closed Genome(s)	Sequence Type	Phage(s) Identified
FSW0104<br>CFSAN087304	ST185	Aeromo_phiO18P_NC009542<br>Salmon_118970_sal3_NC031940
CFSAN000258<br>CFSAN045764<br>CFSAN051296	ST316	Salmon_Fels2_NC010463<br>Salmon_vB_SosS_Oslo_NC_018279
CFSAN005645<br>CFSAN010209	ST138	Salmon_Fels2_NC010463<br>Escher_D108_NC_013594
