# Supplementary material for: Genomic evidence of environmental and resident Salmonella Senftenberg and Montevideo contamination in the pistachio supply-chain
Source: PLoS One. 2021 Nov 4;16(11):e0259471. doi: 10.1371/journal.pone.0259471 (PMC8568146; doi:10.1371/journal.pone.0259471)
Supplement: S7 Table — The direct plating results are shown in this table for triplicate samples taken at different time points throughout the storage study. The water activity measurements taken of these samples is also shown. (PDF) [file pone.0259471.s007.pdf]

S7 Table: Results of storage study of inoculated pistachios stored at 35% and 54% RH sampled at different time points.

<table>
<tr><td></td><td colspan="4">log CFU/g</td><td colspan="2">Water Activity</td></tr>
<tr><td>Day</td><td>Inoculation</td><td>Post-Desiccation</td><td>35% RH</td><td>54% RH</td><td>35% RH</td><td>54% RH</td></tr>
<tr><td>-1</td><td>7.05
7.10
7.08</td><td></td><td></td><td></td><td></td><td></td></tr>
<tr><td>0</td><td></td><td>5.63
5.97
6.08</td><td>5.63
5.97
6.08</td><td>5.63
5.97
6.08</td><td>0.4601</td><td>0.4601</td></tr>
<tr><td>1</td><td></td><td></td><td>5.77
6.07
6.11</td><td>6.39
5.99
6.19</td><td>0.4183</td><td>0.5663</td></tr>
<tr><td>2</td><td></td><td></td><td>5.91
5.90
5.74</td><td>5.95
5.87
5.85</td><td>0.3697</td><td>0.5723</td></tr>
<tr><td>4</td><td></td><td></td><td>5.51
5.61
5.85</td><td>5.76
5.76</td><td>0.3466</td><td>0.5613</td></tr>
<tr><td>6</td><td></td><td></td><td>5.97
5.95
5.51</td><td>6.28
5.72
6.06</td><td>0.3438</td><td>0.5622</td></tr>
<tr><td>13</td><td></td><td></td><td>5.87
5.41
5.68</td><td>5.64
5.77
5.68</td><td>0.4430</td><td>0.5504</td></tr>
<tr><td>27</td><td></td><td></td><td>5.04
5.62
5.44</td><td>5.10
5.13
5.37</td><td>0.3372</td><td>0.5559</td></tr>
<tr><td>55</td><td></td><td></td><td>5.67
5.28
5.41</td><td>4.81
5.37
5.09</td><td>0.3274</td><td>0.5495</td></tr>
<tr><td>83</td><td></td><td></td><td>5.47
5.41
5.23</td><td>4.94
5.18
5.04</td><td>0.3327</td><td>0.5492</td></tr>
<tr><td>168</td><td></td><td></td><td>5.08
5.47
5.43</td><td>4.67
4.46
4.52</td><td>0.3127</td><td>0.5355</td></tr>
<tr><td>251</td><td></td><td></td><td>4.72
4.98
4.98</td><td>3.82
3.45
3.93</td><td>0.2853</td><td>0.5031</td></tr>
<tr><td>365</td><td></td><td></td><td>4.79
4.87
5.21</td><td>2.80
2.72
2.68</td><td>0.3258</td><td>0.5406</td></tr>
</table>
