# Supplementary material for: Genomic evidence of environmental and resident Salmonella Senftenberg and Montevideo contamination in the pistachio supply-chain
Source: PLoS One. 2021 Nov 4;16(11):e0259471. doi: 10.1371/journal.pone.0259471 (PMC8568146; doi:10.1371/journal.pone.0259471)

S8 Figure: Results of the Luminex Serotyping of colonies picked at different time points from inoculated pistachios stored at 35% and 54% RH.

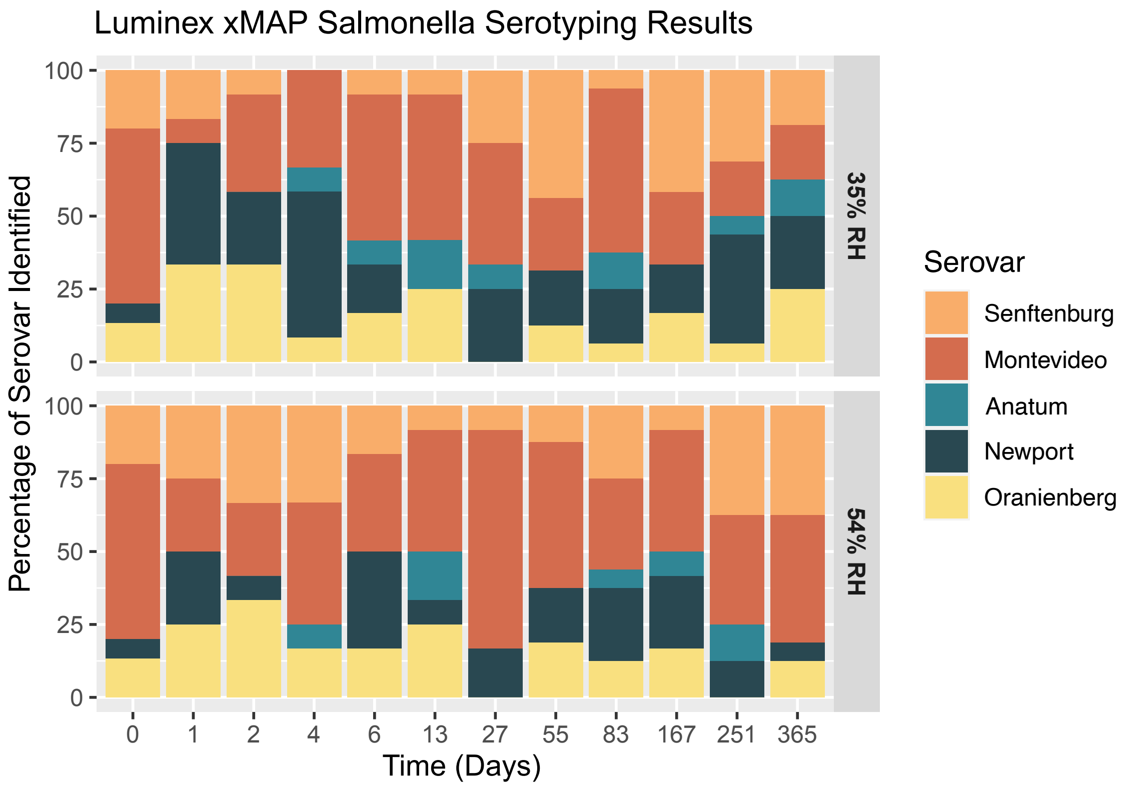

Supplement: S1 Fig — This figure shows the percentage of serotypes identified at different time points from direct plating of inoculated pistachios stored at 35% RH and 54% RH. (PDF) [file pone.0259471.s008.pdf]
